# A Single-Nucleotide Deletion in the Transcription Factor Gene *bcsmr1* Causes Sclerotial-Melanogenesis Deficiency in *Botrytis cinerea*

**DOI:** 10.3389/fmicb.2017.02492

**Published:** 2017-12-12

**Authors:** Yingjun Zhou, Long Yang, Mingde Wu, Weidong Chen, Guoqing Li, Jing Zhang

**Affiliations:** ^1^State Key Laboratory of Agricultural Microbiology and Key Laboratory of Plant Pathology of Hubei Province, Huazhong Agricultural University, Wuhan, China; ^2^Laboratory of Biological Processing, Institute of Bast Fiber Crops, Chinese Academy of Agricultural Sciences, Changsha, China; ^3^United States Department of Agriculture, Agricultural Research Service, Washington State University, Pullman, WA, United States

**Keywords:** *Botrytis cinerea*, orange-colored sclerotia, sclerotial melanogenesis, *bcsmr1*, single-nucleotide deletion

## Abstract

*Botrytis cinerea* is an important plant pathogenic fungus with a wide range of host. It usually produces black-colored sclerotia (BS) due to deposition of 1,8-dihydroxynaphthalene melanin in sclerotial melanogenesis. Our previous study (Zhou et al., 2018) reported six *B. cinerea* isolates producing orange-colored sclerotia (OS) with deficiency in sclerotial melanogenesis. Comparison of ecological fitness (conidia, mycelia, sclerotia), natural distribution, and melanogenesis of selected BS and OS isolates suggests that sclerotia play an important role in the disease cycle caused by *B. cinerea*. However, the molecular mechanism for formation of the OS *B. cinerea* remains unknown. This study was done to unravel the molecular mechanism for the sclerotial melanogenesis deficiency in the OS isolates. We found that all the five sclerotial melanogenesis genes (*bcpks12, bcygh1, bcbrn1/2, bcscd1*) were down-regulated in OS isolates, compared to the genes in the BS isolates. However, the sclerotial melanogenesis-regulatory gene *bcsmr1* had similar expression in both types of sclerotia, suggesting the sclerotial melanogenesis deficiency is due to loss-of-function of *bcsmr1*, rather than lack of expression of *bcsmr1*. Therefore, we cloned *bcsmr1* from OS (*bcsmr1*^*OS*^) and BS (*bcsmr1*^*BS*^) isolates, and found a single-nucleotide deletion in *bcsmr1*^*OS*^. The single-nucleotide deletion caused formation of a premature stop codon in the open reading frame of *bcsmr1*^*OS*^, resulting in production of a 465-aa truncated protein. The transcription activity of the truncated protein was greatly reduced, compared to that of the 935-aa full-length protein encoded by *bcsmr1*^*BS*^ in the BS isolates. The function of *bcsmr1*^*OS*^ was partially complemented by *bcsmr1*^*BS*^. This study not only elucidated the molecular mechanism for formation of orange-colored sclerotia by the spontaneous mutant XN-1 of *B. cinerea*, but also confirmed the regulatory function of *bcsmr1* in sclerotial melanogenesis of *B. cinerea*.

## Introduction

*Botrytis cinerea* is an important plant pathogenic fungus in cool and temperate regions. It can infect more than 1,400 plant species, including about 500 commercially important vegetables, fruits, and ornamentals, causing gray mold disease (Elad et al., 2016). *B. cinerea* usually produces gray-colored conidia and hyphae, and black-colored sclerotia on the surface of the infected plant tissues, as it can synthesize melanin and deposit that dark pigment in the cell wall of hyphae, conidia, and sclerotia (Zeun and Buchenauer, 1985; Doss et al., 2003; Zhang et al., 2015; Schumacher, 2016).

Melanins are chemically diverse and polymerized phenolic or indolic compounds occurring in various organisms such as insects, mammals, plants, and fungi (Bell and Wheeler, 1986; Butler and Day, 1998; Jacobson, 2000; Hamilton and Gomez, 2002). So far, four groups of melanins, namely allomelanins, eumelanins, neuromelanins, and pheomelanins, have been identified based on their chemical structures (Ambrico, 2016). Previous studies have characterized two groups of fungal melanins, namely allomelanins such as 1,8-dihydroxynaphthalene melanin or DHN melanin, and eumelanins such as 3,4-dihydroxyphenylalanine melanin or DOPA melanin (Sapmak et al., 2015; Li et al., 2016).

Melanins are multifunctional compounds (Cordero and Casadevall, 2017). It is well-recognized that melanin deposition in the cell wall can improve fungal survival through enhancing fungal adaptation to environmental stresses, such as UV irradiation, desiccation, enzymatic lysis, toxic chemicals, antagonists, and predators (Bell and Wheeler, 1986; Butler and Day, 1998). In some plant pathogenic fungi, such as *Colletotrichum legenarium* and *Pyricularia oryzae*, melanin is a virulence factor, as it can facilitate appressorium-mediated penetration into plant tissues (Kubo et al., 1982; Howard and Valent, 1996; Chen et al., 2004). In other plant pathogenic fungi, such as *Alternaria brassicicola*, however, melanin biosynthesis was found to be negatively associated with virulence (Cho et al., 2012). In *B. cinerea*, melanin is dispensable for pathogenicity, and instead, it may affect longevity of conidia and sclerotia as proposed in previous studies (Zhang et al., 2015; Schumacher, 2016).

*B. cinerea* and many other ascomycetous fungi produce DHN melanins (Zeun and Buchenauer, 1985; Bell and Wheeler, 1986; Butler and Day, 1998; Doss et al., 2003; Schumacher, 2016). They derive from the polyketide pathway, which starts with the reaction for formation of 1,3,6,8-tetrahydroxynaphthalene (T4HN) from acetate units (precursor) under the catalysis of polyketide synthase or PKS (Bell and Wheeler, 1986; Butler and Day, 1998). Then, T4HN is sequentially converted to scytalone, 1,3,8-trihydoxy-naphthalene (T3HN), vermelone, and DHN monomers through reduction and dehydration reactions. Finally, the DHN monomers are polymerized through oxidative reactions to form the end product DHN melanin.

Previous studies have identified several genes related to melanogenesis in *B. cinerea* (Schumacher et al., 2014; Zhang et al., 2015; Cohrs et al., 2016; Schumacher, 2016). The core genes for the melanogenic enzymes include *bcpks12*/*13* (for two PKSs), *bcygh1* (for a hydrolase), *bcbrn1/2* (for two reductases), and *bcscd1* (for a dehydratase). Moreover, Saitoh et al. (2010) reported that *bcCCC2* coding for a copper-transporting ATPase affects mycelial melanogenesis in *B. cinerea*. The regulatory genes involving melanogenesis of *B. cinerea* include those for transcription factors (TFs) such as *bcsmr1, bcztf1*/*2, bcltf1*/*2, bcvelA, bcvelB*, and *bcvel1* (Schumacher et al., 2013, 2014; Yang et al., 2013a; Cohrs et al., 2016; Schumacher, 2016), and those for signal transduction such as *bcptpA, bcptpB, bcptc1, bos1, bmp3*, and *sak1* (Liu et al., 2011; Yang et al., 2013b,c). Among these genes, *bcsmr1, bcpks12, bcbrn1/2*, and *bcscd1* are required for sclerotial melanogenesis (Schumacher, 2016). Single disruption of *bcsmr1, bcpks12, bcscd1*, and *bcbrn1/2*, and double disruption of *bcpks12*/*13* and *bcbrn1/2* caused deficiency in sclerotial melanogenesis. The disruption-mutants Δ*bcsmr1*, Δ*bcpks12*, and ΔΔ*bcpks12*/*13* produced yellow-colored sclerotia without accumulation of DHN melanin in the epidermal cell wall (Schumacher, 2016).

Sclerotia are generally regarded as the dormancy structure for survival under adverse environmental conditions. Sclerotia produced by *B. cinerea* can germinate to produce conidia. However, importance of the sclerotia as the primary infection source remains controversial in the life cycle of *B. cinerea*, as they are not frequently observed in infected tissues of many diseased plants (Williamson et al., 2007). Zhou et al. (2018) reported six field isolates of *B. cinerea* producing orange-colored sclerotia (OS). The sclerotia of the OS isolate XN-1 were found to be deficient in sclerotial melanogenesis, compared to the black-colored sclerotia produced by isolate B05.10 of *B. cinerea* (Zhou et al., 2018). The orange-colored sclerotia were greatly reduced for survival, compared to the black-colored sclerotia. Meanwhile, Zhou et al. (2018) also found that the OS *B. cinerea* rarely occurred in nature. Therefore, DHN melanin deposition on the sclerotia of *B. cinerea* plays an important role in ecological fitness of sclerotia. However, the molecular mechanism responsible for formation of the orange-colored sclerotia by the field isolates of *B. cinerea* remains unknown. This study was conducted to address this question. Here, we report that a single-nucleotide deletion in *bcsmr1* caused formation of a premature stop codon, resulting in formation of a truncated protein with loss-of-function in regulation of sclerotial melanogenesis.

## Materials and methods

### Fungal isolates and culture media

A total of eight isolates of *B*. *cinerea* were used in this study (Table 1). Among these isolates, XN-1, S417, and T417 are OS isolates, whereas B05.10, HS016, S59, WXto2-2, and XN087 are BS isolates. All these fungal isolates were maintained in 20% glycerol (v/v) at −80°C for the long-term storage. Working cultures of each isolate were prepared by transferring the mycelia of that isolate in storage on potato dextrose agar (PDA) and the resulting cultures were incubated in the dark at 20°C for 5 days. Seven cultural media (MYA, PDA, PDB, PRM, YPDA, SD/-Trp, and SD/-Trp-His) were used in this study. The composition of these media was listed in Table S1.

**Table 1:** Origin of the isolates of *Botrytis cinerea* used in this study.

**Isolate**	**Origin (location, time)**	**Host plant**
**ORANGE-COLORED SCLEROTIAL ISOLATES**		
S417	Xian Tao County, Hubei, China, 2013	Strawberry (*Fragaria* × *ananassa*)
T417	Xiao Chang County, Hubei, China, 2013	Tomato (*Lycopersicon esculentum*)
XN-1	Xian Ning County, Hubei, China, 2012	Unknown
**BLACK-COLORED SCLEROTIAL ISOLATES**		
B05.10	Germany	Table grape (*Vitis vinifera*)
XN087	Xian Ning County, Hubei, China, 2012	Unknown
HS016	Xian Ning County, Hubei, China, 2012	Unknown
WXt02-2	Wu Xue County, Hubei, China, 2013	Tomato (*Lycopersicon esculentum*)
S59	Wuhan, Hubei, China, 2013	Strawberry (*Fragaria* × *ananassa*)

### Gene cloning and sequence analysis

Six *B. cinerea* isolates (XN-1, S417, T417, HS016, WXt02-2, XN087) were separately incubated on cellophane film (CF)-PDA at 20°C for 3 days (Zhou et al., 2018). The mycelial mats were collected from the cultures, and used for extraction of genomic DNA (gDNA) using the CTAB method (Zeng et al., 2014) and the total RNA using the Trizol® reagents (TaKaRa Biotechnol. Co. Ltd., Dalian, China). The gDNA was used as template to PCR-amplify the DNA sequences of *bcsmr1* as well as *bcpks12/13, bcbrn1/2*, and *bcscd1* with the specific primers and thermal programs (Tables S2, S3). The PCR product was separated by agarose gel electrophoresis. The target DNA band in the gel was purified and inserted into the pMD18-T vector (TaKaRa). The resulting re-combined plasmid was subsequently transformed into the competent cells of *E. coli* DH5α. The positive clones harboring the expected DNA insert were sent to AuGCT Biotechnological Company Ltd. (Beijing, China). The nucleotide (nt) sequences for *bcsmr1* (including the promoter region) from different isolates and the amino acid (aa) sequences for BcSMR1 encoded by *bcsmr1* were identified by BLAST searches in the public database (http://www.ncbi.nlm.nih.gov). The characteristic domains for BcSMR1 encoded by *bcsmr1* were identified based on the annotation information of that protein in the reference isolate B05.10. Multiple alignments of the nt sequences of *bcsmr1* or the aa sequences of BcSMR1 in the seven isolates as well as in B05.10 were done using DNAMAN version 5.2.2 (Lynnon Biosoft, Vaudreuil, Quebec, Canada).

### Prokaryotic expression of *bcsmr1*

The full-length cDNA of *bcsmr1* was cloned by reverse transcription (RT)-PCR from the OS isolate XN-1 and the BS isolate B05.10 using the primer pair bcsmr1-etF/bcsmr1-etR (Table S2). The primers contain a *Not* I-restriction site. The resulting cDNA was separated by agarose gel electrophoresis and purified from the gel. Then, the cDNA was digested with *Not* I to release the target fragments of *bcsmr1* from XN-1 (designated as *bcsmr1*^*OS*^) or B05.10 (designated as *bcsmr1*^*BS*^). The two cDNA fragments were separately inserted into the plasmid pET28a(+) (Beijing TransGen Biotechnol. Co. Ltd., Beijing China) at the *Not* I site and two new plasmids, namely pET28a-bcsmr1^OS^ and pET28a-bcsmr1^BS^ (both had a 6× His tag), were then regenerated (Figure S1). The two plasmids were separately transformed into the competent cells of *E. coli* BL21 (DE3) pLysS (Promega, Madison, WI, USA). Positive clones for the two plasmids were screened based on growth on LB containing kanamycin (50 μg/mL). They were shake-incubated and induced for production of the proteins BcSMR1^OS^ and BcSMR1^BS^ using the procedures described by Lou et al. (2015). The bacterial proteins were separated by 8% SDS-PAGE and visualized by staining with Coomassie brilliant blue G-250.

### Western blotting

The proteins in *E. coli* transformed with pET28a-bcsmr1^OS^ or pET28a-bcsmr1^BS^ were prepared using the procedures described by Lou et al. (2015) and were separated by 8% SDS-PAGE. After electrophoresis, the proteins in the gel were transferred by electrophoresis blotting (20 V, 30 min) to a piece of Bio-Rad® polyvinylidene fluoride (PVDF) membrane (6 × 4 cm, length × width) in Trans-Blot® SD Semi-Dry Electrophoretic Transfer Cell. The membrane was washed in TBST buffer (20 mmol/L Tris-base, 150 mmol/L NaCl, 0.05% Tween 20), followed by soaking for 2 h in 5% (w/v) Difco^TM^ skim milk solution alone and then in 5% skim milk solution amended with 0.2% (w/v) anti-His tag antibody (Sangon Biotechnol. Co. Ltd., Shanghai, China) for another 2 h. After that, the membrane was washed again in the TBST buffer for three times, 10 min each time, followed by soaking for 2 h in 5% skim milk solution amended with 0.1% (w/v) alkaline phosphatase-conjugated goat anti-mouse IgG (Sangon Biotechnol. Co. Ltd., Shanghai, China). After another washing in the TBST buffer, the membrane was soaked in 10 mL Western Blue® Stablized Substrate for Alkaline Phosphatase (Promega) to visualize the specific protein bands on the membrane. Finally, the membrane was dipped in water to stop the reaction and photographed to show the hybridzation.

### Gene transcription activation assay

The transcription activity of *bcsmr1*^*OS*^ and *bcsmr1*^*BS*^ was determined using Matchmaker™ GAL4 two-hybrid system 3 (Clontech Laboratories, 2007). The cDNA sequences for *bcsmr1*^*BS*^ in the BS isolate B05.10 (2,080 bp long), *bcsmr1*^*OS*^ in the OS isolate XN-1 (2,079 bp long), and *bcactA* (coding for actin) in B05.10 (1,128 bp long) were separately amplified by RT-PCR with the total RNA extracts from two isolates as templates using the specific RT-PCR primer sets and the specific thermal programs (Tables S2, S3). Meanwhile, the DNA sequence containing the GAL4 activation domain (AD_GAL4_, 408 bp long) was PCR amplified from the plasmid pGADT7 (TaKaRa) with the primer set GAL4-F/GAL4-R (Table S2). The resulting cDNA fragments for *bcsmr1*^*OS*^, *bcsmr1*^*BS*^, and *bcactA*, and the DNA fragment of AD_GAL4_ were purified from a 1% agarose gel after electrophoresis. They were separately inserted into the plasmid pGBKT7 containing the DNA binding domain of GAL4 (BD_GAL4_) using ClonExpress® II One Step Cloning Kit (Vazyme Biotechnol. Co. Ltd., Nanjing, China). The resulting plasmids pBD_GAL4_-AD_GAL4_ (positive control), pGBKT7 (negative control 1), pBD_GAL4_-BcACTA (negative control 2), pBD_GAL4_-BcSMR1^OS^, and pBD_GAL4_-BcSMR1^BS^ were separately transformed into the yeast cells of strain AH109 using the LiAc/SS carrier DNA/PEG transformation method (Clontech Laboratories, 2007). Selection of the yeast mutants harboring the plasmids and detection of their transcription activity were done using the procedures described by Lou et al. (2015).

### Determination of the transcripts of the melanogenesis-related genes

The transcripts of *bcsmr1, bcygh1, bcpks12/13, bcbrn1/2*, and *bcscd1* in the sclerotia of the *B. cinerea* isolates were determined using qRT-PCR with the specific primers and thermal cycles (Tables S2, S3). All the investigated isolates were incubated on PDA at 20°C. The sclerotial primordia, immature sclerotia, and mature sclerotia were collected from the 6-, 8-, and 15-day-old cultures of each isolate, respectively. The total RNA in the sclerotial samples was extracted using TaKaRa RNAiso Reagent Kit (TaKaRa) and was subjected to treatment with DNase I (TaKaRa) to eliminate DNA contamination in the extracts. The treated RNA was then used as templates in qRT-PCR to determine the transcripts of *bcactA* (reference), *bcsmr1, bcpks12/13, bcygh1, bcbrn1/2*, and *bcscd1*. The transcript levels of each gene were calculated by the ^ΔΔ^Ct method (Zeng et al., 2014). For normalization of the data, the transcript level of each gene in the sclerotial primordia of isolate B05.10 was considered as 1.0. The scale was used to calibrate the transcript level of that gene in the immature and mature sclerotia of B05.10, as well as in the sclerotial primordia, immature sclerotia, and mature sclerotia of other isolates. The qRT-PCR assay was independently repeated three times.

### Complementation of the sclerotial melanogenesis in XN-1 with *bcsmr1^*bs*^*

The complementation plasmid pKS1004-BS was constructed based on the plasmid pKS1004 containing the hygromycin-resistance gene (*hph*) (Zeng et al., 2014). The full-length cDNA of *bcsmr1*^*BS*^ from B05.10 was ligated at the downstream of the *Aspergillus nidulans* promoter P*olic* by fusion PCR. The resulting DNA fragment was inserted into the plasmid pKS1004 using the protocol and the reagents in ClonExpress® II One Step Cloning Kit (Vazyme Biotechnol. Co. Ltd., Nanjing, China). The plasmid was then transformed into the protoplasts of XN-1 (Zeng et al., 2014). The transformed protoplasts were regenerated at 20°C on the PRM medium (Table S1) amended with 50 μg/mL hygromycin B. The emerging colonies (isolates) were individually transferred to PDA. Meanwhile, isolates B05.10 and XN-1 were inoculated on PDA as two controls. The cultures (isolates) were incubated at 20°C for 35 days for production of sclerotia. Six complementation mutants were selected based on formation of brown- to black-colored sclerotia (indicating sclerotial melanogenesis). Integration of *bcsmr1*^BS^ in the genomes of the complementation mutants was confirmed by PCR using the primer set Olic-F/bcsmr1R (Table S2). XN-1 was used as reference in the specific PCR assay. Two representative complementation mutants, namely XN-BS11 and XN-BS14, were selected for determination of the copy number of *bcsmr1* by Southern blotting also with XN-1 as a reference. The *bcsmr1-*specific DNA probe was generated by PCR from the genomic DNA of B05.10 with the primer set bcsmr1-jcF/bcsmr1R (Table S2). In Southern blotting, the gDNA of XN-1, XN-BS11, and XN-BS14 was extracted and digested with *Eco*RI. The resulting DNA fragments were separated by electrophoresis and detected by the DNA probe, which was labeled with the reagents in DIG High Prime DNA Labeling and Detection Starter Kit II (Roche®, Mannheim, Germany).

Expression the six melanogenic genes (*bcpks12*/*13, bcygh1, bcbrn1/2, bcscd1*) in sclerotia produced by XN-1 (negative control), XN-BS11, XN-BS14, and B05.10 (positive control) was determined using qRT-PCR. The procedures for isolation of the total RNA from the sclerotial samples and qRT-PCR were similar to those described above.

### Data analysis

The procedure UNIVARIATE in the SAS software (SAS Institute, Cary, NC, USA, v. 8.0, 1999) was used to analyze the data on relative expression levels of each gene. Average values of each gene between the OS (3 isolates) and BS (4 isolates) groups, and between each complementation mutant and XN-1/B05.10 were compared using Student's *t*-test at *P* < 0.05 or *P* < 0.01.

## Results

### Expression of the genes for sclerotial melanogenesis is suppressed in the orange-colored sclerotia, compared to that in the black-colored sclerotia

**Figure 1:**
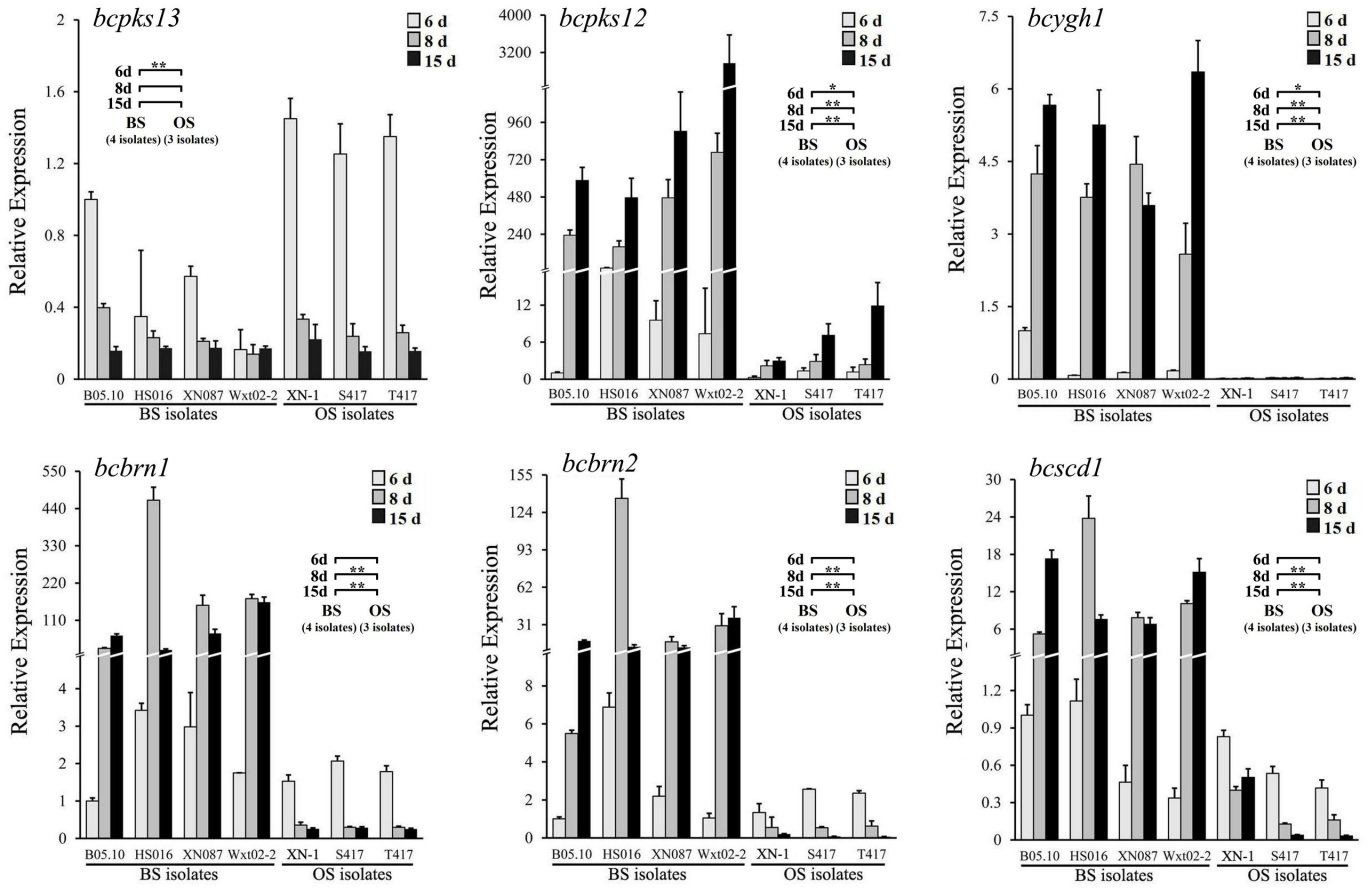
Relative expression values of six melanogenic enzyme-coding genes (*bcpks12/13, bcygh1, bcbrn1/2*, and *bcscd1*) in the sclerotia of *B. cinerea* isolates. The sclerotial primordia, immature sclerotia, and mature sclerotia of each isolate were collected from 6-, 8-, and 15-day-old PDA cultures (20°C), respectively. The results are the representative of three independent tests. Bars indicate standard deviations of the means. In each graph, “*” at *P* < 0.05 and “**” at *P* < 0.01 between the OS and BS isolates (as two groups) according to Student's *t*-test.

To analyze the melanin-biosynthesis ability in orange-colored sclerotia of *B. cinerea*, expression of six melanogenic enzymes-coding genes, including *bcpks12/13, bcygh1, bcbrn1/2*, and *bcscd1*, in the sclerotia of seven isolates of *B. cinerea* (Table 1) was determined by qRT-PCR using the specific primers and thermal programs (Tables S2, S3). The results showed that all the seven investigated isolates (OS isolates: XN-1, S417, T417; BS isolates: B05.10, HS016, XN087, WXt02-2) had low expression levels of *bcpks13* at all sclerotial developmental stages (Figure 1). Thus, *bcpks13* did not seem to be involved in the formation of the orange-colored sclerotia.

However, significant differences were found between the OS and BS isolates in expression levels of the polyketide synthase gene *bcpks12* (Figure 1). Although all the seven investigated isolates showed the increased expression pattern in maturation of sclerotia, the expression levels were significantly lower in the OS isolates than in the BS isolates. In the sclerotial primordia (white-colored) from the 6-day-old PDA cultures (20°C), the expression level of *bcpks12* was low with the relative expression values (REVs) ranging from 0.3 to 1.4 for the OS isolates and from 1.0 to 22.6 for the BS isolates. In the immature sclerotia (gray-colored) from the 8-day-old PDA cultures and in the mature sclerotia (black- and orange-colored in the BS and OS isolates, respectively) from 15-day-old PDA cultures, the *bcpks12* expression levels slightly increased by 2.6 and 7.8-folds, respectively, in the OS isolates, whereas were largely increased by 40.4 and 121.8-folds, respectively, in the BS isolates (Figure 1).

For the other sclerotial melanogenesis genes (*bcygh1, bcbrn1/2, bcscd1*), the OS isolates differed from the BS isolates both in expression pattern and in expression level (Figure 1). While the BS isolates showed an increased expression pattern, the OS isolates showed a decreased expression pattern. In the sclerotial primordia of the OS isolates, the REVs ranged from 0.01 to 0.03 for *bcygh1*, 1.5 to 2.1 for *bcbrn1*, 1.3 to 2.6 for *bcbrn2*, and 0.4 to 0.8 for *bcscd1*. In the sclerotial primordia of the BS isolates, the REVs ranged from 0.1 to 1.0 for *bcygh1*, 1.0 to 3.4 for *bcbrn1*, 1.0 to 6.9 for *bcbrn2*, and 0.3 to 1.1 for *bcscd1*. In the immature sclerotia and mature sclerotia, expression of these genes was decreased by 43.2–88.5% in the OS isolates, whereas it was increased by 8 to 90-folds in the BS isolates, compared the corresponding REVs of these genes in the sclerotial primordia of B05.10.

To rule out the possibility that suppressed expression of *bcpks12, bcygh1, bcbrn1/2*, and *bcscd1* in the sclerotia of the OS isolates is caused by mutations of these genes, the DNA sequences of these genes were cloned from the BS and OS isolates mentioned above (Table S4). Alignment analysis showed that the identity of the DNA sequences of each gene and the amino acid sequences of each protein in the seven above-mentioned isolates of *B. cinerea* was higher than 98% (Table S5). Single-amino acid polymorphisms were detected in the amino acid sequences of each protein (Figure S2). However, none of the polymorphic amino acids was found to be associated with formation of the orange-colored sclerotia. Therefore, suppressed expression of *bcpks12, bcygh1, bcbrn1/2*, and *bcscd1* in the sclerotia of the OS isolates of *B. cinerea* is probably not caused by the point mutations in these genes. It might be caused by some regulatory genes for the sclerotial melanogenesis.

### The OS isolates have a single-nucleotide deletion in *bcsmr1*

**Figure 2:**
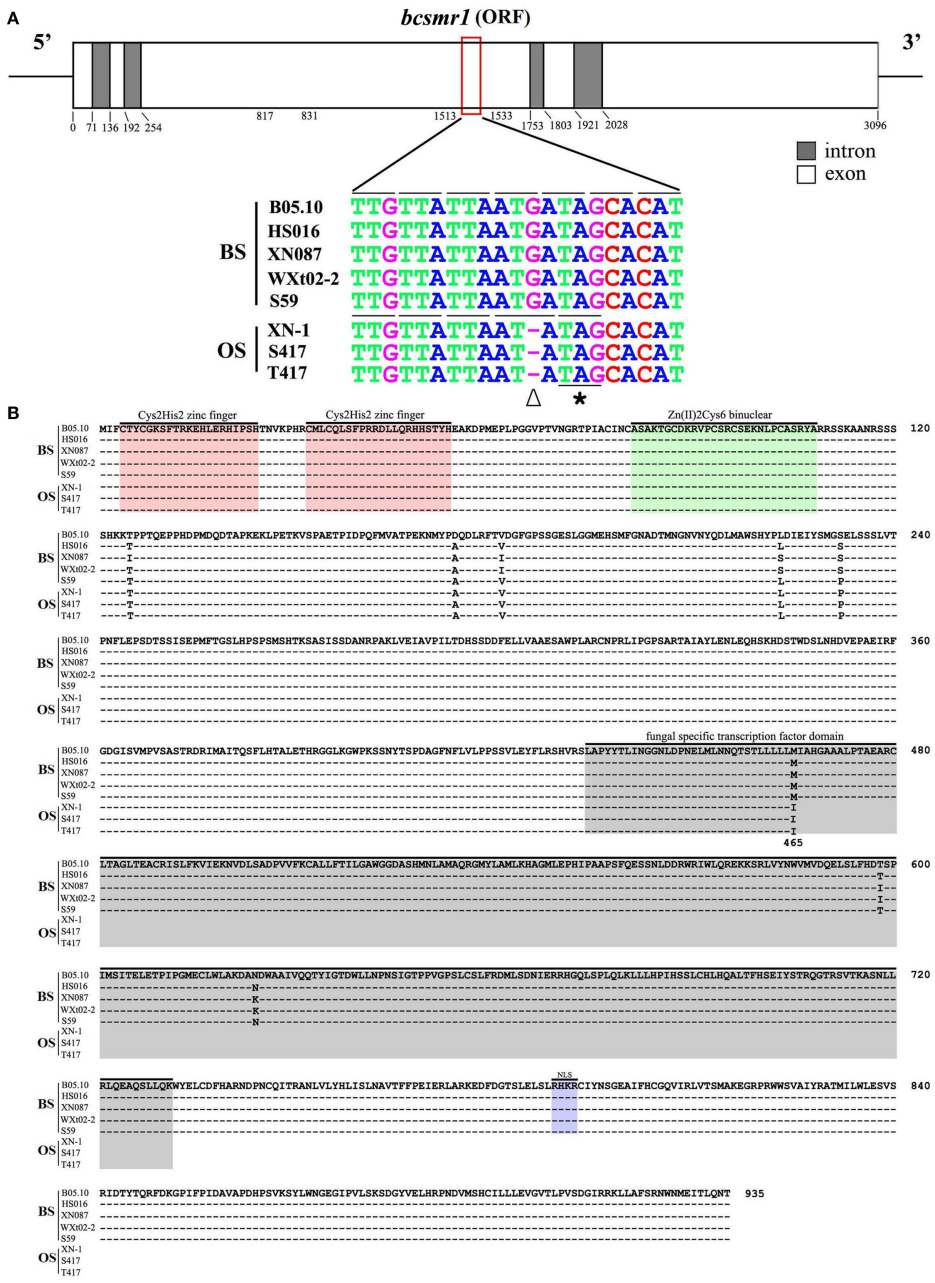
Comparison of the nucleotide sequences of the transcription factor gene *bcsmr1* in *Botrytis cinerea* and the amino acids sequences encoded by *bcsmr1*. **(A)** A schematic diagram showing the structure of *bcsmr1* and alignment of the partial DNA sequences of *bcsmr1* in different isolates. Nucleotide positions for the exons and introns are indicated. OS, orange-colored sclerotia, BS, black-colored sclerotia. The symbols “∇” and “*” indicate the single-nucleotide deletion and the in-frame stop codon, respectively; **(B)** Alignment of the deduced amino acid sequences of BcSMR1 in different isolates. The symbol “–” in each column indicates the identical amino acids. Note difference in length of BcSMR1 in the OS and BS isolates, 465 aa long for BcSMR1 in the OS isolates, whereas 935 aa long for BcSMR1 in the BS isolates. NLS, Nuclear Localization Signaling region.

To confirm whether the abnormal expression pattern of the melanin biosynthesis genes was caused by some regulatory malfunction, the transcription factor (TF) gene *bcsmr1* related to the melanin biosynthesis was cloned from three OS isolates (XN-1, S417, T417) and four BS isolates (HS016, S59, WXt02-2, XN087) by PCR using the specific primers and specific thermal cycles (Tables S2, S3). The DNA sequences for *bcsmr1* in these isolates were submitted to GenBank and were assigned with the accession numbers from KU743104 to KU743109, and KX098785 (Table S4). They were aligned with the DNA sequence of *bcsmr1* in the reference isolate B05.10 (Accession Number: Bcin02g08760 in the website http://fungi.ensembl.org/Botrytis_cinerea). The results showed that *bcsmr1* in the OS isolates (designated as *bcsmr1*^*OS*^) differed from *bcsmr1* in other five BS isolates (designated as *bcsmr1*^*BS*^) in length of the open reading frame (ORF), 3,096 bp long for *bcsmr1*^*BS*^, whereas 3,095 bp long for *bcsmr1*^*OS*^ (Figure 2A). The single-nucleotide deletion in *bcsmr1*^*OS*^ is located at the position of nt 1,524, where guanine (G) is present in *bcsmr1*^*BS*^, but absent in *bcsmr1*^*OS*^. Thus, the single-nucleotide deletion was designated as G1524X. In spite of the single-nucleotide deletion, the identity of the DNA sequences in the ORF region of *bcsmr1*^*OS*^ and *bcsmr1*^*BS*^ in the seven isolates was higher than 98% (Table S5).

The single-nucleotide deletion in *bcsmr1*^*OS*^ caused change of the triplet codon at the position nt 1,522 to 1,524 from ATG (for methionine or M) in *bcsmr1*^*BS*^ to ATA (for isoleucine or I) in *bcsmr1*^*OS*^. The subsequent triplet codon at the position nt 1,525 to 1,527 was also changed from ATA (for isoleucine or I) in *bcsmr1*^*BS*^ to TAG (stop) in *bcsmr1*^*OS*^ (Figure 2A). As a consequence, the ORF of *bcsmr1*^*OS*^ was deduced to encode a protein with 465 amino acid (aa) residues (designated as BcSMR1^OS^). It is a truncated protein of the 935-aa protein encoded by the ORF of *bcsmr1*^*BS*^ (designated as BcSMR1^BS^). Alignment analysis showed that both BcSMR1^465^ and BcSMR1^935^ have the DNA binding domain at the N-terminus, consisting of two Cys_2_His_2_-zinc finger motifs and a Zn_2_Cys_6_-binuclear motif (Figure 2B). BcSMR1^OS^ has a small portion (32 aa long) of the fungal specific transcription factor domain at the C-terminus without the nuclear localization signaling, whereas BcSMR1^BS^ has the full fungal specific TF domain (298 aa long) with the nuclear localization signaling region. Alignment analysis also showed that there are six point mutations (T125I, A174D, V181I, L223S, S232P, M465I) in BcSMR1^BS^ and BcSMR1^OS^ (Figure 2B) and five of these mutations (T125I, A174D, V181I, L223S, S232P) were found in both BcSMR1^BS^ and BcSMR1^OS^. Therefore, they are probably not associated with formation of the orange-colored sclerotia. The other mutation, namely M465I, was detected only in BcSMR1^OS^, but it was not detected in BcSMR1^BS^. This mutation is caused by the single-nucleotide-deletion-mediated one-nucleotide shift (Figure 2A). Therefore, the single-nucleotide deletion is probably important for formation of the orange-colored sclerotia by the OS isolates of *B. cinerea*. In spite of the point mutations, the identity of *bcsmr1*^OS^ and *bcsmr1*^BS^ is higher than 99% (Table S5).

**Figure 3:**
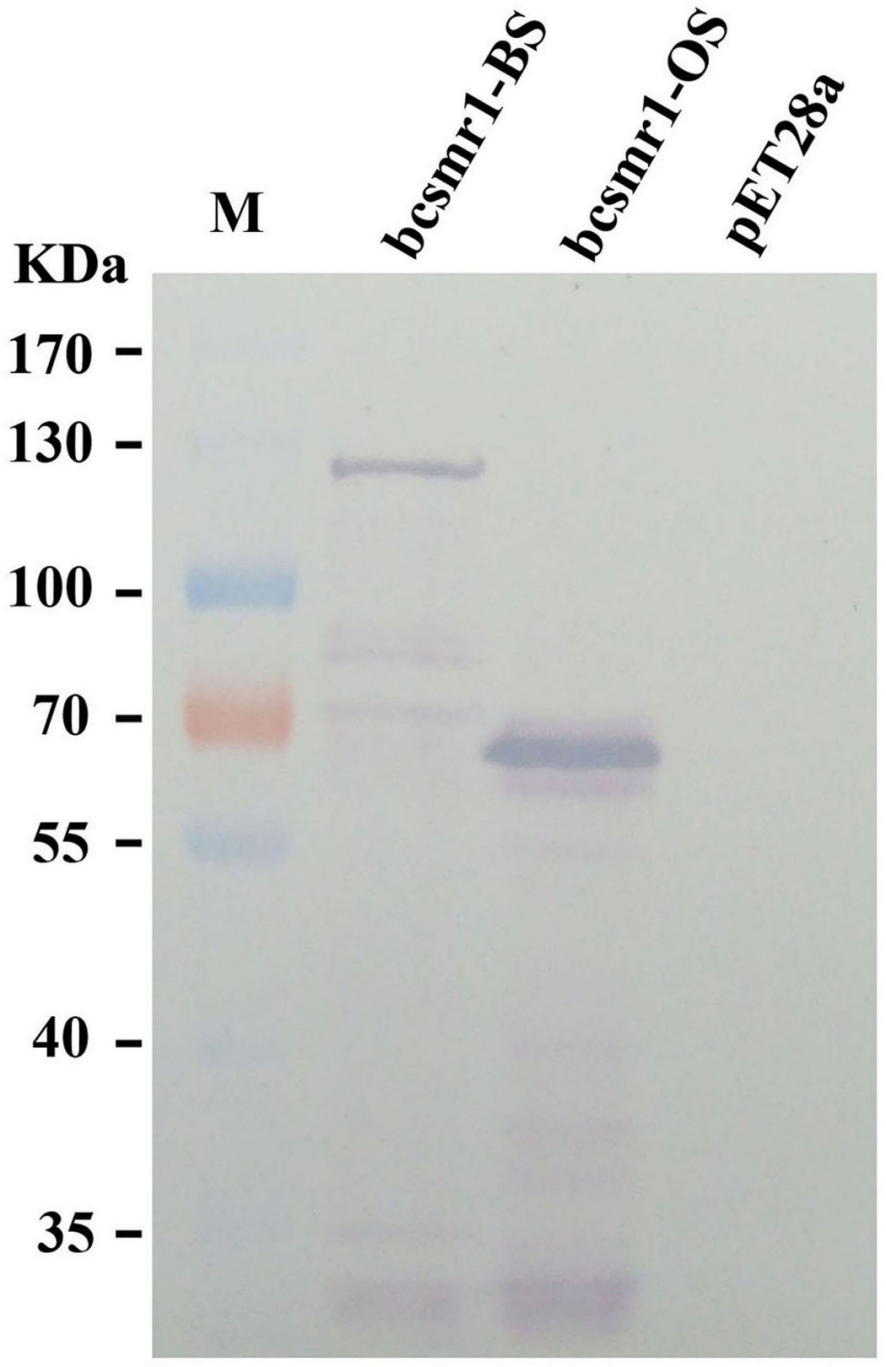
Western blotting detection of the *E. coli* proteins encoded by *bcsmr1*^*BS*^ and *bcsmr1*^*OS*^ using the anti-His tag antibody. The treatments “bcsmr1-BS”and “bcsmr1-OS” represent the proteins encoded by *bcsmr1*^*BS*^, and *bcsmr1*^*OS*^, respectively. The treatment “pET28a” represents the protein encoded by the blank plasmid pET28a alone without *bcsmr1*^*BS*^ or *bcsmr1*^*OS*^. M = PageRuler^TM^ prestained protein ladder.

Prokaryotic expression of *bcsmr1*^*OS*^ and *bcsmr1*^*BS*^ in *E. coli* was done to confirm formation of BcSMR1^OS^ through the single-nucleotide deletion-mediated truncation of BcSMR1^BS^. The full-length cDNA of *bcsmr1*^*OS*^ in XN-1 and *bcsmr1*^*BS*^ in B05.10 were separately cloned by RT-PCR using the specific primers and specific thermal program (Tables S2, S3). The resulting cDNA fragments were separately introduced into the expression vector pET28a(+) (Figure S1). The resulting two plasmids, namely pET28a-bcsmr1^OS^ and pET28a-bcsmr1^BS^, were then constructed and separately transformed into *E. coli* BL21 (DE3) pLysS. The transformed bacterial clones were selected, followed by shake-incubation in LB medium for production of BcSMR1^OS^ and BcSMR1^BS^ under induction by isopropyl β-D-1-thiogalactopyranoside (IPTG). The bacterial proteins extracted from different *E. coli* cultures were detected by 8% SDS-PAGE. Results showed that two differentially-expressed proteins were consistently detected in the positive transformants of *E. coli* (data not show). The molecular weight was ~66 and 120 kD (both had a 6× His tag) in the transformants with pET28a-bcsmr1^OS^ and pET28a-bcsmr1^BS^, respectively. The 54-kD difference in molecular weight between the two proteins is close to the 53.2-kD difference in molecular weight between the putative BcSMR1^OS^ and BcSMR1^BS^, which had the predicted molecular weight of 51.3 and 104.5 kD, respectively. Western blotting analysis using the anti-His tag antibody further confirmed production of the 66- and 120-kD proteins by *bcsmr1*^*OS*^ and *bcsmr1*^*BS*^, respectively, in *E. coli* (Figure 3).

### The transcription activity of *bcsmr1*^OS^ is greatly suppressed, compared to that of *bcsmr1^*Bs*^*

**Figure 4:**
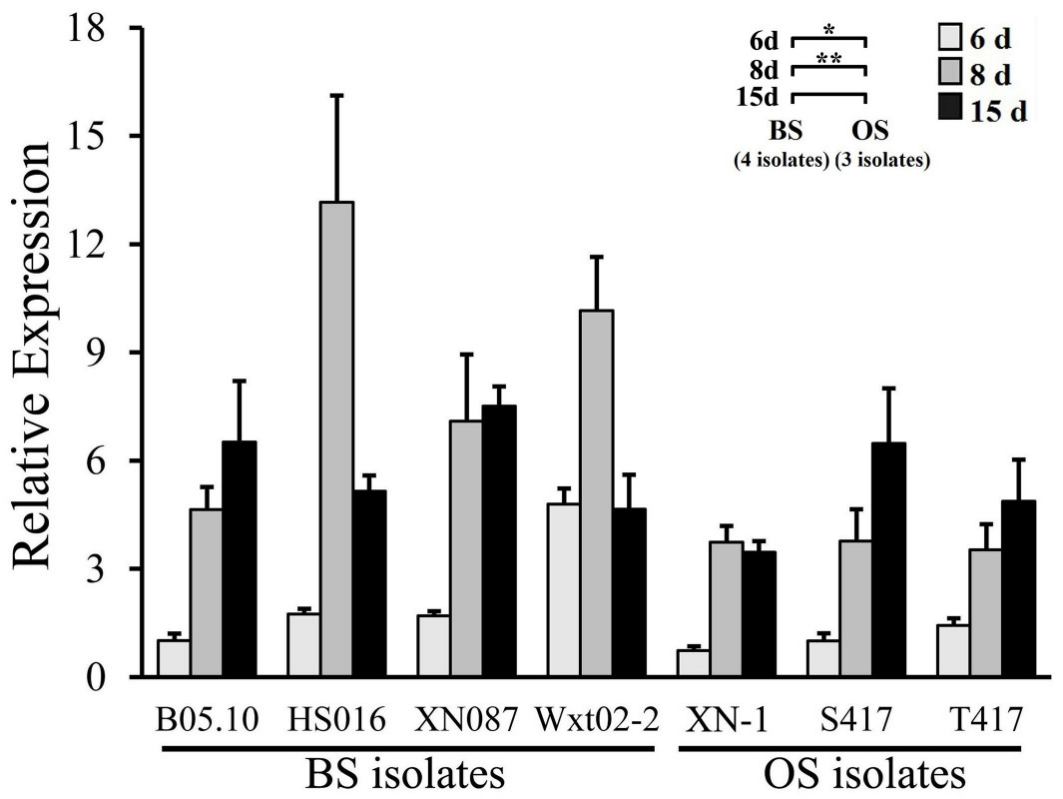
Relative expression values of the transcription factor gene *bcsmr1* in the sclerotia of *B. cinerea*. The sclerotial primordia, immature sclerotia, and mature sclerotia of each isolate were collected from 6-, 8-, and 15-day-old PDA cultures (20°C), respectively. The results are the representative of the three independent tests. Bars indicate standard deviations of the means. “*” at *P* < 0.05 and “**” at *P* < 0.01 between the OS and BS isolates (as two groups) according to Student's *t*-test.

**Figure 5:**
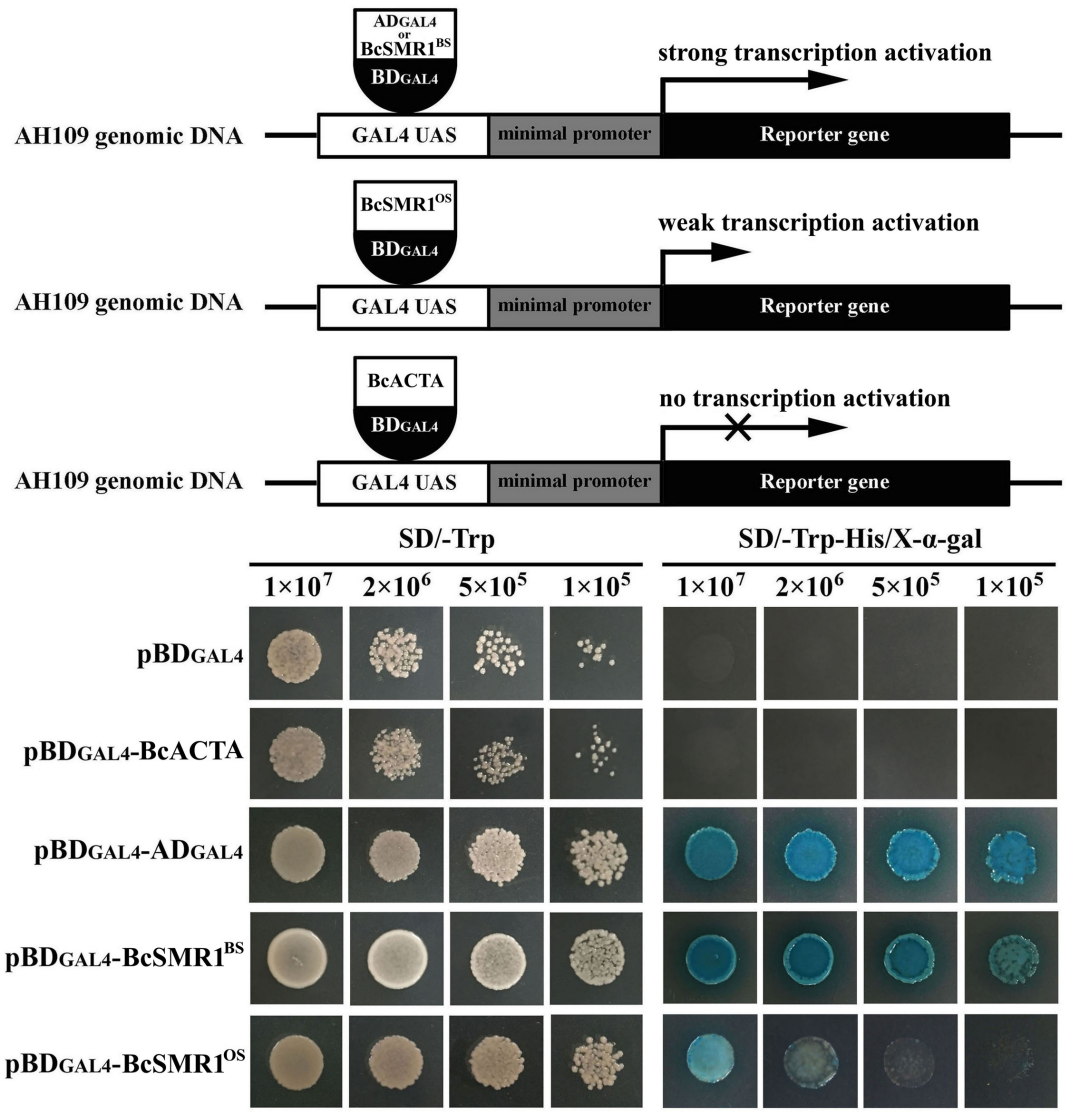
Transcription activation of *bcsmr1* in *Saccharomyces cerevisiae* AH109. **(Top)** Three schematic diagrams depicting the experimental principle. AD_GAL4_ and BD_GAL4_ stand for the GAL4 activation domain and the GAL4 DNA-binding domain, respectively. BcSMR1^BS^ and BcSMR1^OS^ stand for the proteins encoded by *bcsmr1*^*BS*^ from the BS isolate B05.10 and *bcsmr1*^*OS*^ from the OS isolate XN-1, respectively. BcACTA stands for the protein encoded by *bcactA* from B05.10. BcSMR1^BS^, BcSMR1^OS^, and AD_GAL4_ can initiate transcription of the reporter genes *HIS3, ADE2*, and *MEL1*; **(Bottom)** Difference between *bcsmr1*^*BS*^ and *bcsmr1*^*OS*^ in transcription activity. Compared to AD_GAL4_, BcSMR1^BS^ initiated strong gene transcription, whereas BcSMR1^OS^ initiated weak gene transcription. Growth of the yeast mutants on SD/-Trp (left panel, 30°C, 1 day) indicates complementation of the auxotrophic mutant AH109. Growth of the yeast mutants on SD/-Trp-His (right panel, 30°C, 1 day) and production of blue color on the yeast colonies in the presence of X-α-Gal indicate the positive activity of gene transcription.

Both the OS isolates and the BS isolates had an increased expression pattern of *bcsmr1* in sclerotia (Figure 4). We hypothesized that compared to *bcsmr1*^*BS*^, *bcsmr1*^*OS*^ might be suppressed for activating transcription of the downstream genes for sclerotial melanogenesis. To test this hypothesis, the transcription activity of *bcsmr1*^*OS*^ and *bcsmr1*^*BS*^ was determined in *Saccharomyces cerevisiae* AH109 using Matchmaker™ GAL4 Two Hybrid System 3 (Clontech Laboratories, 2007). Five plasmids (pBD_GAL4_, pBD_GAL4_-BcACTA, pBD_GAL4_-AD_GAL4_, pBD_GAL4_-BcSMR1^OS^, and pBD_GAL4_-BcSMR1^BS^) were constructed. Four plasmids (pBD_GAL4_-BcACTA, pBD_GAL4_-AD_GAL4_, pBD_GAL4_-BcSMR1^OS^, and pBD_GAL4_-BcSMR1^BS^) contain two domains, namely the DNA-binding domain (e.g., BD_GAL4_) and the activation domain (e.g., AD_GAL4_, BcACTA, BcSMR1^OS^, BcSMR1^BS^). The remaining plasmid pBD_GAL4_ contains BD_GAL4_ alone. The plasmids were separately transformed into the yeast cells and the resulting transformants were screened on two auxotrophic media SD/-Trp and SD/Trp-His. On SD/-Trp, all the mutants showed growth. On SD/-Trp-His, however, the mutants differed in growth (Figure 5). While the transformants harboring either pBD_GAL4_ or pBD_GAL4_-BcACTA failed to grow on SD/-Trp-His, the transformants of pBD_GAL4_-AD_GAL4_, pBD_GAL4_-BcSMR1^OS^, and pBD_GAL4_-BcSMR1^BS^ grew on this medium and produced blue color on the colonies when X-α-gal was added. This result indicates that AD_GAL4_, *bcsmr1*^*BS*^, and *bcsmr1*^*OS*^ can activate transcription of the MEL1 gene coding for α-galactosidase. Relatively, the transformants of pBD_GAL4_-AD_GAL4_ and pBD_GAL4_-BcSMR1^BS^ grew better than the transformant harboring pBD_GAL4_-BcSMR1^OS^. The blue color on the colonies of the transformants of pBD_GAL4_-AD_GAL4_ and pBD_GAL4_-BcSMR1^BS^ was darker than that on the colonies of the transformant of pBD_GAL4_-BcSMR1^OS^. Therefore, compared to *bcsmr1*^*BS*^, *bcsmr1*^*OS*^ is greatly suppressed for transcription of *MEL1*.

### Complementation of *bcsmr1^*OS*^* with *bcsmr1^*BS*^* restored sclerotial melanogenesis in XN-1

**Figure 6:**
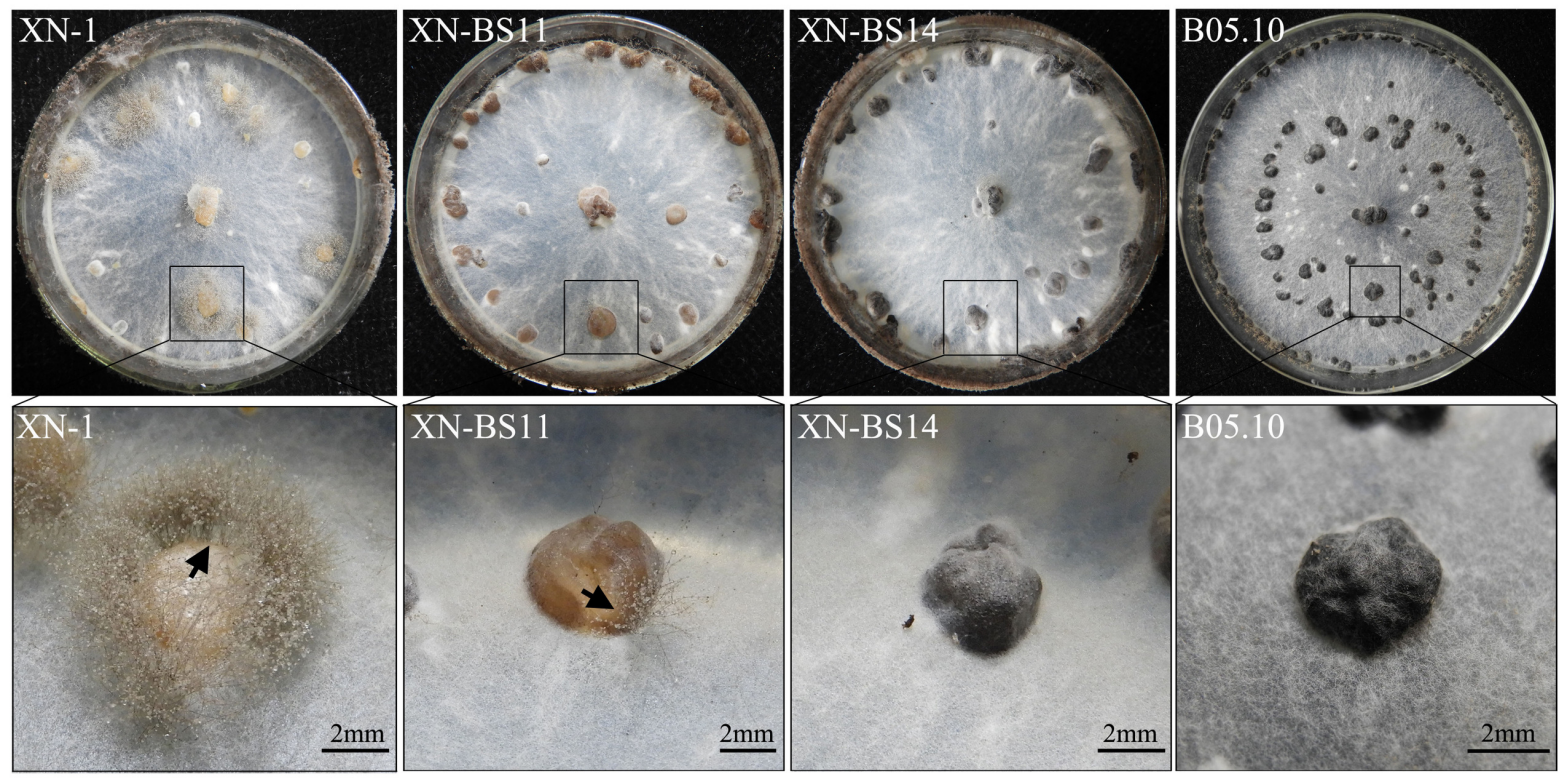
Colony morphology of *Botrytis cinerea* isolates B05.10 (BS), XN-1 (OS) and two complementation mutants of XN-1 (XN-BS11, XN-BS14) on potato dextrose agar after incubation at 20°C for 35 days. Note color of the sclerotia formed by different isolates. The arrow mark indicates sporogenic germination on the sclerotia.

To confirm responsibility of the single-nucleotide deletion in *bcsmr1*^*OS*^ for sclerotial melanogenesis deficiency, the full-length cDNA of *bcsmr1*^*BS*^ from B05.10 was used to complement the function of *bcsmr1*^*OS*^ in XN-1. A complementation plasmid pKS1004-BS containing the cDNA of *bcsmr1*^*BS*^ under the control of the constitutive *A. nidulans* P*oliC* promoter was successfully constructed on the basis of the plasmid pKS1004 harboring the hygromycin-resistance gene *hph* (Zeng et al., 2014). The plasmid was introduced into XN-1 by protoplast-mediated transformation. Ten transformants were obtained based on hygromycin resistance. After incubation at 20°C on PDA for 35 days, XN-1 formed orange-colored sclerotia, as it has defect in sclerotial melanogenesis, whereas B05.10 formed black-colored sclerotia, as it has normal sclerotial melanogenesis (Figure 6). Four of these transformants formed orange-colored sclerotia like those formed by XN-1, indicating that the sclerotial melanogenesis in these transformants was not restored at all. In contrast, six other transformants (XN-BS1, XN-BS2, XN-BS11, XN-BS12, XN-BS14, XN-BS16) formed partially-melanized sclerotia, which were characterized by formation of color mosaic on the sclerotial surface (Figure 6). These six transformants could be classified into two groups based on the color mosaic on the sclerotia. Three transformants (XN-BS11, XN-BS12, XN-BS16) showed a brown-gray mosaic pattern, whereas the other three transformants (XN-BS1, XN-BS2, XN-BS14) showed a gray-black mosaic pattern. This result indicates that the sclerotial melanogenesis in these transformants was at least partially restored, compared to the complete sclerotial melanogenesis in B05.10 and the defective sclerotial melanogenesis in XN-1. Meanwhile, sporogenic germination (indicating lack of dormancy) was observed on the orange sclerotia produced by XN-1 and on the brown-grayish sclerotia produced by the transformants XN-BS11, XN-BS12, and XN-BS16 (Figure 6). The sporulation appeared to be vigorous on the sclerotia of XN-1, whereas was sparse on the sclerotia of the three transformants. In contrast, the black-colored sclerotia produced by B05.10 and the gray-colored sclerotia produced by the transformants XN-BS1, XN-BS2, and XN-BS14 did not germinate at all. This result indicates that the dormancy deficiency of the orange sclerotia (germination-active) of XN-1 due to lack of melanin was partially restored in the partially-melanized sclerotia produced by the complementation mutants.

The six complementation transformants were confirmed for integration of *bcsmr1*^*BS*^ in their genomes by PCR using the primer set Olic-F/bcsmr1R (Table S2) (data not shown). Two of these transformants (XN-BS11, XN-BS14) were further determined for *bcsmr1*^*BS*^ integration in their genomes by Southern blotting using the *bcsmr1*-specific DNA probe (Figure S3A). XN-1 was used as the reference isolate in Southern blotting. The result showed that the three isolates had one to three hybridization bands, one band in XN-1, two bands in XN-BS11, and three bands in XN-BS14 (Figure S3B). This result indicates that the genomes of XN-1, XN-BS11, and XN-BS14 harbor one (*bcsmr1*^*OS*^), two (*bcsmr1*^*OS*^ + *bcsmr1*^*BS*^), and three (*bcsmr1*^*OS*^ + *bcsmr1*^*BS*^ + *bcsmr1*^*BS*^) copies of *bcsmr1*, respectively.

Expression of the six melanogenic genes (*bcpks12/13, bcygh1, bcbrn1/2, bcscd1*) in the sclerotia of isolates XN-1, XN-BS11, and XN-BS14 was determined by qRT-PCR. The two transformants were similar to XN-1 both in expression pattern and in expression level of *bcpks13*, which is not required for sclerotial melanogenesis according to Schumacher (2016). All the three isolates showed a decreased expression pattern and low expression levels of *bcpks13* in sclerotial maturation.

**Figure 7:**
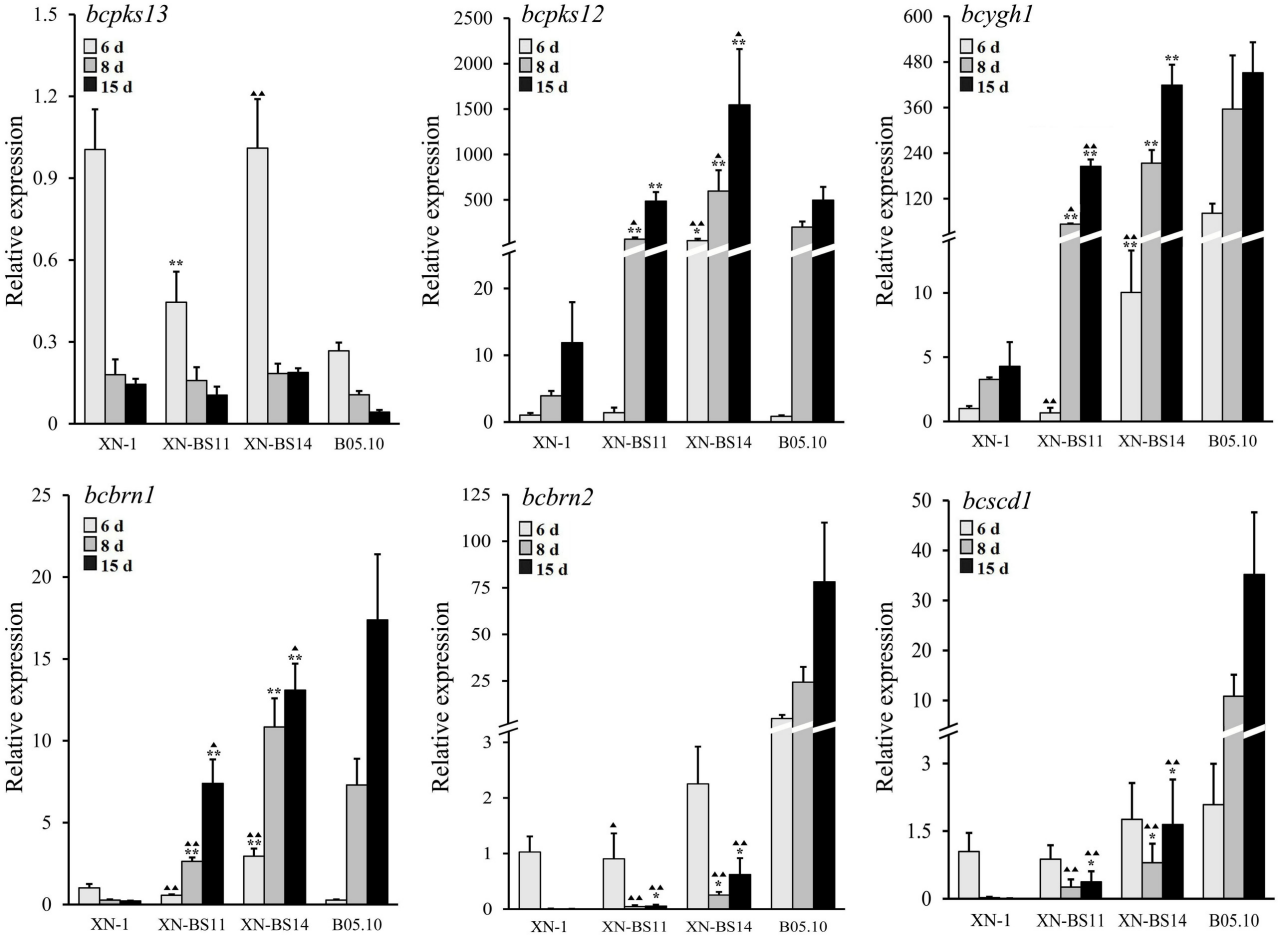
Relative expression values of the six melanogenic enzyme-coding genes (*bcpks12/13, bcygh1, bcbrn1/2, bcscd1*) in the sclerotia of isolate XN-1 of *Botrytis cinerea*, two complemented mutants of XN-1 (XN-BS11, XN-BS14) and B05.10. The sclerotial primordia, immature sclerotia, and mature sclerotia of each isolate were collected from 6-, 8-, and 15-day-old PDA cultures (20°C), respectively. The results are the representative of three independent tests. Bars indicate standard deviations of the means. ^*^P < 0.05 and ^**^P < 0.01 (Student's *t*-test) between each mutant and XN-1; 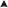 *P* < 0.05 and 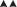 *P* < 0.01 (Student's *t*-test) between each mutant and B05.10.

In contrast, the two transformants differed greatly from XN-1 either in expression pattern or in expression level of the other five sclerotial melanogenesis genes (*bcpks12, bcygh1, bcbrn1/2, bcscd1*). For *bcpks12* and *bcygh1*, the two transformants were similar to XN-1 in expression pattern, but differed from XN-1 in expression level (Figure 7). The two genes showed an increased expression pattern in maturation of the sclerotia produced by the three isolates. In the sclerotial primordia, the REVs ranged from 1.0 to 49.8 for *bcpks12* and from 1.0 to 10.0 for *bcygh1*. In the immature sclerotia and mature sclerotia, the REVs of the two genes were largely increased by 12 to 350-folds in the two transformants, whereas were slightly increased by 3 to 12-folds in XN-1.

For *bcbrn1*, the two transformants differed from XN-1 both in expression pattern and in expression level (Figure 7). In XN-1, the REV was reduced from 1.0 in the sclerotial primordia to < 0.3 in the immature sclerotia/mature sclerotia. In XN-BS11 and XN-BS14, however, the REVs were increased in maturation of the sclerotia. XN-BS11 had the REV increase from 0.6 in the sclerotial primordia to 2.6 in the immature sclerotia, and 7.4 in mature sclerotia. XN-BS14 had a REV increase from 2.9 in the sclerotial primordia to 10.8 in the immature sclerotia, and 13.1 in the mature sclerotia.

For *bcbrn2* and *bcscd1*, the two transformants were also similar to XN-1 in expression pattern, but differed from XN-1 in expression level (Figure 7). All the three isolates showed a decreased expression pattern in maturation of the sclerotia. In the sclerotial primordia, the three isolates had the REVs of 0.9–2.3 and 0.9–1.8 for *bcbrn2* and *bcscd1*, respectively. In the immature sclerotia and mature sclerotia, while XN-1 had negligible expression of both genes, XN-BS11 had the REVs of 0.1–0.6 for *bcbrn2* and 0.4–1.7 for *bcscd1*. In general, XN-BS14 had higher REVs of *bcpks12, bcygh1, bcbrn1/2*, and *bcscd1* than the corresponding REVs for XN-BS11 (Figure 7). Meanwhile, the expression pattern of *bcpks12, bcygh1*, and *bcbrn1/2* in the mutants XN-BS11 and XN-BS14 resembled that in the BS isolate B05.10, although the expression levels of these genes at each stage were significantly (*P* < 0.05 or *P* < 0.01) lower than those of B05.10 (Figure 7). This result further confirmed that the single-nucleotide deletion in *bcsmr1* is the major reason for formation of the orange-colored sclerotia.

## Discussion

Zhou et al. (2018) first reported natural occurrence of orange-colored sclerotial (OS) isolates of *B. cinerea* infecting strawberry and tomato in central China. The OS isolates are natural mutants or variants of *B. cinerea*, compared to the normal isolates with formation of black-colored sclerotia. Microscopic observation showed that both orange- and black-colored sclerotia of *B. cinerea* are quite similar in structure, consisting of the cortex layer (outside) with compact parenchymatous cells and the medulla (inside) with loose prosenchymatous cells. However, the sclerotia of the two types differ greatly in melanin deposition in the wall of the epidermal cells. While the BS isolates have normal melanin deposition in the black-colored sclerotia, the OS isolates are deficient in melanin deposition in the orange-colored sclerotia (Zhou et al., 2018). Our previous study also showed that the OS isolates occur rarely in nature, as the sclerotia produced by the OS isolates of *B. cinerea* survived poorly, compared to the sclerotia produced by the BS isolates (Zhou et al., 2018). Therefore, mutation of the sclerotial color from black to orange greatly changed the ecological fitness of *B. cinerea*.

Zeun and Buchenauer (1985) reported that *B. cinerea* produces DHN melanin, as tricyclazole, an inhibitor for DHN melanin biosynthesis, can delay sclerotial melanogenesis in *B. cinerea*. Schumacher (2016) elucidated the molecular pathway for biosynthesis of DHN melanin in *B. cinerea*. The result showed that *bcpks12* is required for sclerotial melanogenesis, *bcpks13* is required for conidial melanogenesis, and *bcbrn1/2* and *bcscd1* are required for both sclerotial melanogenesis and conidial melanogenesis (Schumacher, 2016). The present study revealed that the OS isolates differed greatly from the BS isolates either in expression pattern or in expression level of *bcpks12, bcygh1, bcbrn1/2*, and *bcscd1*. The expression levels of these genes in the immature and mature sclerotia of the three OS isolates were significantly lower than those of the four BS isolates (Figure 1). This molecular evidence corroborates our previous study in microscopic observation that the orange-colored sclerotia was due to lack of melanin deposition (Zhou et al., 2018).

A recent study by Schumacher (2016) showed that the transcription factor (TF) gene *bcsmr1* regulates the sclerotial melanogenesis of *B. cinerea*. This gene has only one copy in isolate B05.10 of *B. cinerea* (Schumacher, 2016). The Δ*bcsmr1* mutant formed yellow-colored sclerotia on agar media, indicating lack of sclerotial melanogenesis (Schumacher, 2016). *Bcsmr1* is an ortholog of *CMR1* (*Colletotrichum Melanin Regulation 1*) in *Collectrichum lagenarium* (Tsuji et al., 2000). It is also homologous to other orthologs of *CMR1*, including *Amr1* and *CmrA* in *Alternaria* species (Cho et al., 2012; Fetzner et al., 2014), *BMR1* in *Bipolaris oryzae* (Kihara et al., 2008), *ChCMR1* in *Cochliobolus heterostrophus* (Eliahu et al., 2007), and *PIG1* in *Magnaporthe grisea* (Sweigard et al., 1998).

*Bcsmr1* and other *CMR1* orthologs are similar in structure. They contain a DNA-binding domain with two Cys_2_His_2_ zinc finger motifs and one Zn(II)_2_Cys_6_ binuclear cluster motif at the N-terminus, and a fungal specific transcription factor (TF) domain and a nuclear localization signaling (NSL) region at the C-terminus (Figure 2B). Tsuji et al. (2000) found that the DNA-binding domain in *CMR1* is necessary for melanization in *C. lagenarium*. Fetzner et al. (2014) reported that CmrA encoded by *CmrA* can bind to the promoter region of *pksA* (coding for polyketide synthase), thereby initiating expression of *pksA* and the subsequent melanogenesis in *A*. *alternata*.

This study demonstrated that both BS and OS isolates of *B. cinerea* had an increased expression pattern of *bcsmr1* in the sclerotia (Figure 4). The result is consistent with the finding of Schumacher (2016), who found that expression of *bcsmr1* is associated with sclerotial development of *B. cinerea*. It implies that failure to regulate the sclerotial melanogenesis by *bcsmr1*^*OS*^ in the OS isolates of *B. cinerea* is not due to expression of this gene. Instead, it may be caused by loss-of-function of *bcsmr1*^*OS*^.

In order to elucidate the mechanism for the loss-of-function of *bcsmr1*^*OS*^, we cloned *bcsmr1*^*OS*^ and *bcsmr1*^*BS*^ from selected isolates in this study, and compared the DNA sequences of *bcsmr1*^*OS*^ with those of *bcsmr1*^*BS*^. Three OS isolates (XN-1, S417, T419) have a single-nucleotide deletion in *bcsmr1*^*OS*^ (Figure 2B). Occurrence of single-nucleotide deletions has been frequently reported in animals (Persuy et al., 1999; Yang et al., 2003) and plants (Takano et al., 2014; Lou et al., 2015), whereas it has been rarely reported in fungi, including *B. cinerea* (Pihet et al., 2009).

Previous studies readily indicated that *B. cinerea* can mutate in response to extreme environments such as presence of UV irradiation and fungicides (Leroux et al., 2002; Kretschmer et al., 2009). These mutagens usually cause formation of single-nucleotide polymorphisms (SNPs) in populations of *B*. *cinerea* (Kretschmer et al., 2009; Schumacher et al., 2012, 2013). Most SNPs in certain genes such as *mbc1* coding for β-tubulin have been found to be closely associated with sensitivity or resistance to fungicides (Leroux et al., 2002; Kretschmer et al., 2009). A few SNPs in other genes such as *bcvel1* coding for the VELVET protein cause formation of a premature stop codon, thereby resulting in production of truncated proteins and change of some important phenotypes such as virulence and sclerotial formation (Schumacher et al., 2012, 2013). Whether or not these mutagens can cause formation of the single-nucleotide deletion in *bcsmr1* remains unknown. Taking together all the information about the low occurrence frequency of the OS isolates of *B. cinerea* in nature and the reduced ecological fitness of the OS isolates, we can draw a conclusion that the single-nucleotide deletion observed in this study is important for *B. cinerea*. Understanding of the mutagens for formation of that single-nucleotide deletion in *bcsmr1* will be helpful for designing a novel strategy for control of *B. cinerea*.

This study demonstrated that the single-nucleotide deletion in *bcsmr1*^*OS*^ caused formation of a premature stop codon, which results in production of a truncated 465-aa protein BcSMR1^OS^ (Figures 2, 3). In contrast, the full-length *bcsmr1*^*BS*^ in the BS isolates codes for a 935-aa protein BcSMR1^BS^ (Figures 2, 3). BcSMR1^OS^ contains the complete DNA-binding domain, but has only the partial transcription factor domain without the nuclear signaling localization region. In *S. cerevisiae* AH109, *bcsmr1*^*OS*^ had reduced activity in activating transcription of the MEL1 gene, compared to *bcsmr1*^*BS*^ (Figure 5). This result suggests that *bcsmr1*^*OS*^ cannot be fully functional in activating transcription of the sclerotial melanogenesis genes in *B. cinerea*. Like other transcription factors, *bcsmr1* is transcribed in the nuclei and the resulting transcripts are translocated into the cytoplasm, where they are translated to the protein BcSMR1. Without the nuclear signaling localization domain, BcSMR1^OS^ may not be so easy as BcSMR1^BS^ in re-entrance into the nuclei from the cytoplasm. Without the full transcription factor domain, BcSMR1^OS^ may not be so fully functional as BcSMR1^BS^ in activating transcription of the downstream genes for sclerotial melanogenesis even it enters into the nuclei somehow. As a consequence, transcription of the sclerotial melanogenesis genes regulated by BcSMR1^OS^ is reduced or blocked, compared to BcSMR1^BS^. Thus, the sclerotial melanin biosynthesis in the OS isolates of *B. cinerea* is suppressed, thereby causing formation of the orange-colored sclerotia.

Introduction of the full-length *bcsmr1*^*BS*^ from the BS isolate B05.10 partially complemented the loss-of-function of *bcsmr1*^*OS*^ in the OS isolate XN-1. Six complementation mutants formed partially-melanized sclerotia (Figure 6). The mutants XN-BS11 and XN-BS14 showed enhanced expression of *bcpks12, bcygh1, bcbrn1/2*, and *bcscd1* in the immature and mature sclerotia in comparison to their progenitor XN-1(Figure 7), confirming the previous finding by Schumacher (2016) that *bcsmr1* has the regulatory function for the sclerotial melanogenesis in *B. cinerea*. Ectopic complementation with the full-length *bcsmr1*^*BS*^ increased expression of *bcpks12, bcygh1*, and *bcbrn1* in sclerotia and the expression pattern was similar to that found in the BS isolate B05.10. However, this study also found that the complementation had negligible effects on expression of *bcbrn1/2* and *bcscd1* (Figure 7). These results suggest that *bcsmr1* has different regulatory effects on expression of these genes. Coincidently, *bcbrn2* and *bcscd1* along with *bcpks13* form one gene cluster (in chromosome No. 3) located at a different chromosomal position from the other sclerotial melanogenesis genes. *Bcsmr1, bcpks12*, and *bcygh1* form a gene cluster located at chromosome No. 2, and *bcbrn1* is located at chromosome No. 4 (Schumacher, 2016). Difference in chromosomal position for *bcsmr1, bcbrn2*, and *bcscd1* may be responsible for the low regulatory efficiency of *bcsmr1* for *bcbrn2* and *bcscd1*. Further studies are necessary to determine the activity of *bcsmr1*^*BS*^ in activating transcription of different sclerotial melanogenesis genes.

This study used the full-length *bcsmr1* from the BS isolate B05.10, instead of the deleted nucleotide, to restore the phenotype of formation of black-colored sclerotia by the OS isolate XN-1. This complementation strategy may mask the possible effects of the point mutations of *bcsmr1* on formation of orange-colored sclerotia. A few point mutations were identified in *bcsmr1* among the eight isolates of *B. cinerea* (5 BS isolates and 3 OS isolates) (Figure 2). However, none of the point mutations was found to be associated with formation of the orange-colored sclerotia. Therefore, the result from the complementation experiment in this study at least strengthened importance of the single-nucleotide deletion in loss-of-function of *bcsmr1* for sclerotial melanogenesis. Future studies are necessary to complement *bcsmr1*^*OS*^ with the deleted nucleotide.

Schumacher (2016) reported that *bcygh1* involves in conidial melanogenesis of *B. cinerea*, as BcYGH1 encoded by *bcygh1* can convert the product catalyzed by BcPKS13 encoded by *bcpks13*, which is required only for conidial melanogenesis. Fan et al. (2017) reported that *Vayg1*, an ortholog of *bcygh1*, is required for microsclerotia formation and melanogenesis of microsclerotia in *Verticillium dahlia*, the causal agent of verticillium wilt of cotton. In this study, we found that like the essential genes for sclerotial melanogenesis (*bcpks12, bcbrn1/2, bcscd1*), *bcygh1* was greatly suppressed for expression in the orange sclerotia of XN-1, compared to expression of *bcygh1* in the black sclerotia of B05.10 (Figure 1). However, when *bcsmr1*^*BS*^ was introduced into XN-1, *bcygh1* expression was greatly increased (Figure 7). This result implies that besides *Bcltf1/2* (two transcription factors), *bcsmr1* may regulate expression of *bcygh1*. It also suggests that the expression of *bcygh1* is closely associated with sclerotial melanogenesis in *B. cinerea*. Further studies are necessary to clarify the interaction between *bcsmr1* and *bcygh1* and to elucidate how the interaction affects the sclerotial melanogenesis in *B. cinerea*.

## Author contributions

YZ and JZ: designed research; LY and MW: provided new agents and analyzed the data; YZ, WC, and GL: wrote the paper.

### Conflict of interest statement

The authors declare that the research was conducted in the absence of any commercial or financial relationships that could be construed as a potential conflict of interest.

## Supplementary material

Figure S1 A schematic diagram showing the procedure for construction of the plasmids used for prokaryotic expression of bcsmr1^OS^ and bcsmr1^BS^ from *Botrytis cinerea* isolates XN-1 and B05.10, respectively.

Figure S2 A schematic diagram showing the structure of the five melanogenic enzymes. **(A)** The proposed pathway for biosynthesis of DHN-melanin in *B. cinerea*. It was modified on the basis of Figure S8 prepared by Schumacher et al. (2014); **(B)** Five schematic diagrams showing differential amino acids in the five melanogenic enzymes among the investigated isolates of *B. cinerea*. Two polyketide synthases: BcPKS12 and BcPKS13 encoded by *bcpks12* and *bcpks13*, respectively; two reductases: BcBRN1 and BcBRN2 encoded by *bcbrn1* and *bcbrn2*, respectively; and one dehydratase: BcSCD1 encoded by *bcscd1*. Abbreviations for the conserved domains: KS, β-ketoacyl synthase domain; AT, acyl transferase domain; ACP, acyl carrier protein domain; TE, thioesterase domain; ENR, enoyl-ACP reductase doamin; SD, scytalone dehydratase domain. The GenBank accession numbers for the polypeptides-coding genes were listed in Table S4.

Figure S3 Southern blotting detection of *bcsmr1* in isolate XN-1 (OS) of *Botrytis cinerea* and two complementation mutants of XN-1 (XN-BS11, XN-BS14). **(A)** Two schematic diagrams showing the position of the *bcsmr1*-specific DNA probe on the DNA sequence of bcsmr1^OS^ and on the complemented cDNA sequence of bcsmr1^BS^; **(B)** Southern blotting detection of *bcsmr1* in different isolates.

Table S1 Cultural media used in this study and their compositions.

Table S2 Oligonucleotides PCR primers used in this study.

Table S3 Thermal programs for PCR and qRT-PCR in this study.

Table S4 The GenBank accession numbers for the melanin biosynthesis-related genes and their promoter regions in different isolates of *B. cinerea*.

Table S5 Identity of *bcsmr1* (open reading frame) and the polypeptide BcSMR1 encoded by *bcsmr1* in black-colored sclerotial (or BS) or orange-colored sclerotial (or OS) isolates of *Botrytis cinerea*.
