# Supplementary figures and images for: A Single-Nucleotide Deletion in the Transcription Factor Gene bcsmr1 Causes Sclerotial-Melanogenesis Deficiency in Botrytis cinerea
Source: Front Microbiol. 2017 Dec 12;8:2492. doi: 10.3389/fmicb.2017.02492 (PMC5733056; doi:10.3389/fmicb.2017.02492)

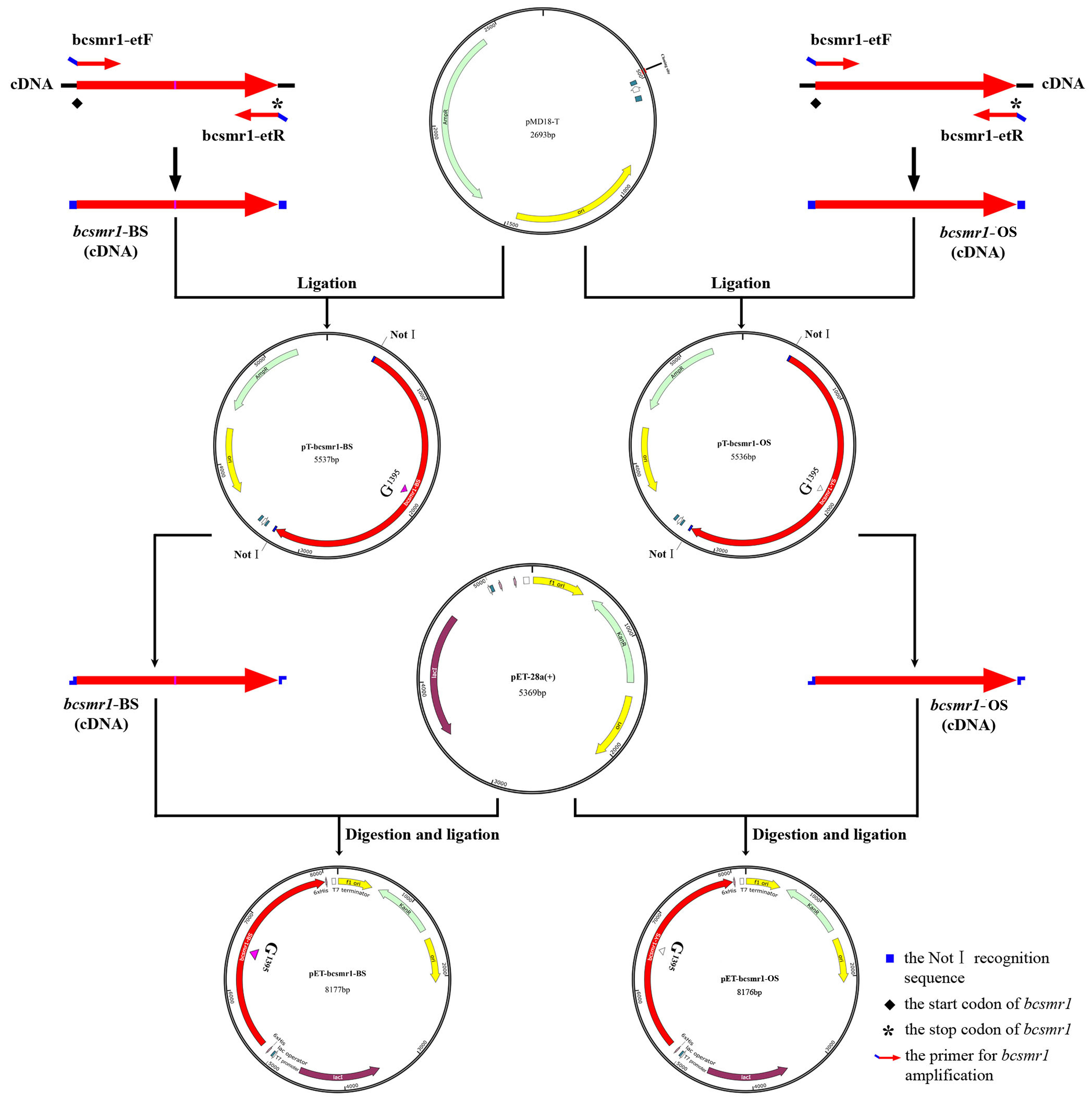

Supplement: Figure S1 — A schematic diagram showing the procedure for construction of the plasmids used for prokaryotic expression of bcsmr1OS and bcsmr1BS from Botrytis cinerea isolates XN-1 and B05.10, respectively. [file Image1.JPEG]

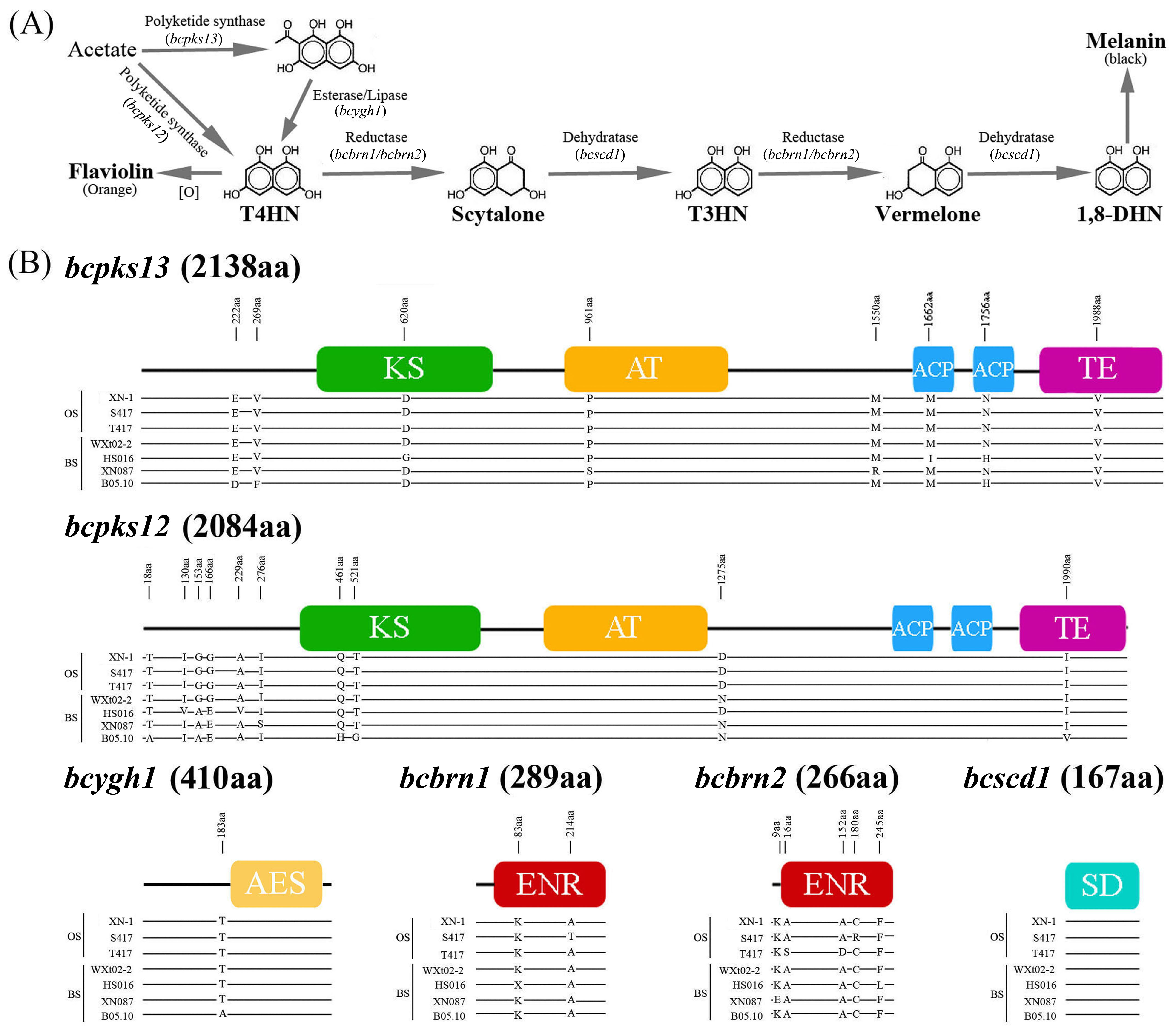

Supplement: Figure S2 — A schematic diagram showing the structure of the five melanogenic enzymes. (A) The proposed pathway for biosynthesis of DHN-melanin in B. cinerea. It was modified on the basis of Figure S8 prepared by Schumacher et al. (2014); (B) Five schematic diagrams showing differential amino acids in the five melanogenic enzymes among the investigated isolates of B. cinerea. Two polyketide synthases: BcPKS12 and BcPKS13 encoded by bcpks12 and bcpks13, respectively; two reductases: BcBRN1 and BcBRN2 encoded by bcbrn1 and bcbrn2, respectively; and one dehydratase: BcSCD1 encoded by bcscd1. Abbreviations for the conserved domains: KS, β-ketoacyl synthase domain; AT, acyl transferase domain; ACP, acyl carrier protein domain; TE, thioesterase domain; ENR, enoyl-ACP reductase doamin; SD, scytalone dehydratase domain. The GenBank accession numbers for the polypeptides-coding genes were listed in Table S4. [file Image2.JPEG]

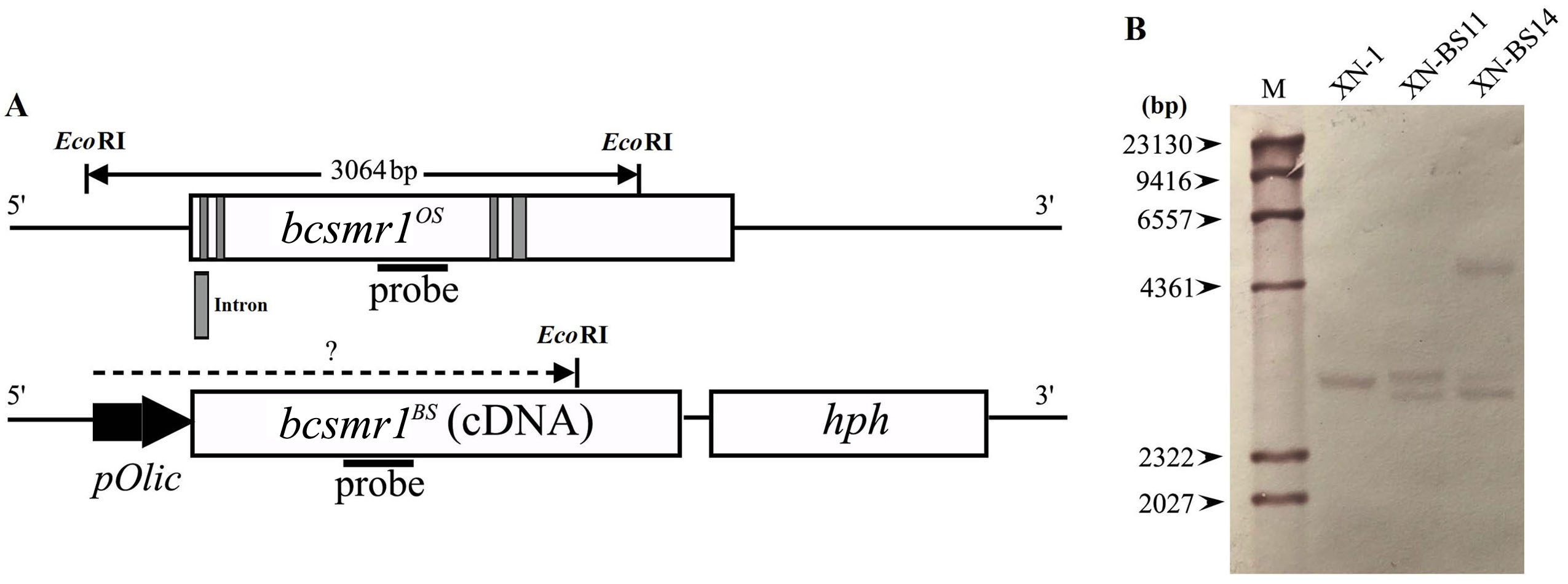

Supplement: Figure S3 — Southern blotting detection of bcsmr1 in isolate XN-1 (OS) of Botrytis cinerea and two complementation mutants of XN-1 (XN-BS11, XN-BS14). (A) Two schematic diagrams showing the position of the bcsmr1-specific DNA probe on the DNA sequence of bcsmr1OS and on the complemented cDNA sequence of bcsmr1BS; (B) Southern blotting detection of bcsmr1 in different isolates. [file Image3.JPEG]
