# Supplementary material for: A Single-Nucleotide Deletion in the Transcription Factor Gene bcsmr1 Causes Sclerotial-Melanogenesis Deficiency in Botrytis cinerea
Source: Front Microbiol. 2017 Dec 12;8:2492. doi: 10.3389/fmicb.2017.02492 (PMC5733056; doi:10.3389/fmicb.2017.02492)
Supplement: Table S1 — Cultural media used in this study and their compositions. [file Table1.DOC]

**Table S1** Cultural media used in this study and their compositions.

| Medium | Composition (in l,000 mL distilled water) | Reference |
| --- | --- | --- |
| MYA | 20 g malt extract, 5 g yeast extract, 12.5 g agar powder | Walker *et al*. (2011) *Phytopathology* **101**: 1433–1445 |
| PDA | Water extract of 200 g peeled potato tubers, 20 g D-glucose, 20 g agar | Ren *et al*. (2007) *Biological Control* **43**: 1–11 |
| PDB | Peeled potato tubers 200 g, 20 g D-glucose | Ren *et al*. (2007) *Biological Control* **43**: 1–11 |
| PRM | 274 g sucrose, 5 g yeast extracts, 2 g bacto-casitone,  8 g agar | Lou *et al*. (2015) *Environmental*  *Microbiology* **17**: 4711–4729 |
| YPDA | 20g peptone, 10g yeast extract, 20g D-Glucose | Lou *et al*. (2015) *Environmental*  *Microbiology* **17**: 4711–4729 |
| SD/-Trp | 26.7g Minimal SD Base, 0.72g -Trp Do supplement, 12,5g agar powder | Lou *et al*. (2015) *Environmental*  *Microbiology* **17**: 4711–4729 |
| SD/-Trp-His | 26.7g Minimal SD Base, 0.62g -Leu-Trp-His Do supplement,0.1g L-leucine, 12,5g agar powder | Lou *et al*. (2015) *Environmental*  *Microbiology* **17**: 4711–4729 |

Note: Abbreviation for the cultural media: MYA = malt-yeast-agar medium; PDA = potato dextrose agar; PDB = potato dextrose broth; PRM = protoplast regeneration agar medium.
