# Supplementary material for: A Single-Nucleotide Deletion in the Transcription Factor Gene bcsmr1 Causes Sclerotial-Melanogenesis Deficiency in Botrytis cinerea
Source: Front Microbiol. 2017 Dec 12;8:2492. doi: 10.3389/fmicb.2017.02492 (PMC5733056; doi:10.3389/fmicb.2017.02492)
Supplement: Table S2 — Oligonucleotides PCR primers used in this study. [file Table2.DOC]

**Table S2** Oligonucleotides PCR primers used in this study.

| Primer | | Sequence (5’→ 3’) | Purpose and reference |
| --- | --- | --- | --- |
| Bcsmr1-1F | | ATGATTTTTTGCACATATTGCGG | PCR cloning of *bcsmr1* (based on the annotated ORF Bcin02g08760) |
| Bcsmr1-1R | | CTATGTGTTCTGCAATGTGATTTCC |
| Bcsmr1-etF | | AAGGAAAAAAGCGGCCGCATGATTTTTTGCACATATTGCGG (*Not* I-restriction site underlined) | Cloning of the cDNA of *bcsmr1* for prokaryotic expression (based on the annotated ORF Bcin02g08760) |
| Bcsmr1-etR | | AAGGAAAAAAGCGGCCGCCTATGTGTTCTGCAATGTGATTTCC (*Not* I-restriction site underlined) |
| Bcpks12-2F | | CATTCATACCGTCGTAAGTGAGC | qRT-PCR detection of the expression of *bcpks12* (based on the annotated ORF Bcin02g08770) |
| Bcpks12-2R | | CTTATAGCCGTACTCGCATAAAGTC |
| Bcpks13-2F | | TCAACCACACGGACCATATC | qRT-PCR detection of the expression of *bcpks13* (Liu *et al*., 2011) |
| Bcpks13-2R | | CGCCATCACCAAGAAGACC |
| Bcygh1-1F  Bcygh1-1R | | CATCTCAAGCAACGGAACTCG  GTCGCCAGAAGCCACTAACTC | qRT-PCR detection of the expression of *bcygh1*(based on the annotated ORF Bcin02g04360) |
| Bcbrn1-2F | | ACAAGAAGATTACCGTCAACTG | qRT-PCR detection of the expression of *bcbrn1* (Liu *et al*., 2011) |
| Bcbrn1-2R | | ACAACTCTGGCAATATCAATAGG |
| Bcbrn2-2F | | GGAACTCGGTAGCAAGGGAT | qRT-PCR detection of the expression of *bcbrn2* (based on annotated ORF Bcin03g08100) |
| Bcbrn2-2R | | TTGGCAGCGATGGAAGAC |
| Bcscd1-2F | | TGCCCGCTTCCCAATTCAT | qRT-PCR detection of the expression of *bcscd1* (based on the annotated ORF Bcin03g08110) |
| Bcscd1-2R | | CCATCACCCTCATTCCATCTTAC |
| Bcsmr1F/  Bcsmr1R | | ACCGTCCAGCCAAACTCG  TCTCGGCTGGTTCCACATC | qRT-PCR detection of expression of *bcsmr1* (based on Bcin02g08760) |
| bcact-F | | TCCAAACCGCCAGTCAATCC | qRT-PCR detection of the expression of *bcactA* (Liu *et al*., 2011) |
| bcact-R | | GATACCACCGCTCTCAAGACC |
| T7 promoter primer | | TAATACGACTCACTATAGGG | Beijing TransGen Biotechnology Co. Ltd., Beijing, China |
| Smr1-bsBD-F | TCAGAGGAGGACCTGCATATGATGATTTTTTGCACATATTGC | | PCR cloning of the full-length  cDNA sequences of *bcsmr1* |
| Smr1-bsBD-R | TCGACGGATCCCCGGGAATTCCTATGTGTTCTGCAATGTGAT | |
| ActinABD-F | TCAGAGGAGGACCTGCATATGATGGAAGAAGAAGTCGCAGCC | | PCR cloning of the full-length cDNA sequence of *bcactA* |
| ActinABD-R | TCGACGGATCCCCGGGAATTCTTAGAAACACTTGCGGTGGAC | |
| GAL4-F | TCAGAGGAGGACCTGCATATGATGGATAAAGCGGAATTAATTC | | PCR cloning of the DNA sequence for the GAL4 activation domain |
| GAL4-R | TCGACGGATCCCCGGGAATTCCTCTTTTTTTGGGTTTGGTGG | |  |
| Bcsmr1-jcF | CTGTTGATGGCTTTGGACCTT | | Generation of the *bcsmr1*-specific DNA probe used in Southern blotting. The primers were designed based on the annotated ORF of *bcsmr1* (Bcin02g08760) in B05.10 |
| Bcsmr1-1R | CTATGTGTTCTGCAATGTGATTTCC | |
| Olic-F  Olic-R | GACGAGCCGCATTCCCGA  CCGCAATATGTGCAAAAAATCATGGATCGATTGTGATGTGATGGAG | | PCR cloning of the constitutive promoter *p*Olic |

References: (1) Liu *et al*. (2011) *Fung. Genet. Biol.* **48**: 377–387; (2) The DNA sequence Bcin02g08760 for *Bcsmr1* was downloaded from the website http://fungi.ensembl.org /Botrytis_cinerea/Info/Index; (3) Clontech Laboratories (2007) Matchmaker™ GAL4 two hybrid system 3 and libraries user manual. Protocol No. PT3247-1, Version No. PR742219, Mountain View, California, USA.
