# Supplementary material for: A Single-Nucleotide Deletion in the Transcription Factor Gene bcsmr1 Causes Sclerotial-Melanogenesis Deficiency in Botrytis cinerea
Source: Front Microbiol. 2017 Dec 12;8:2492. doi: 10.3389/fmicb.2017.02492 (PMC5733056; doi:10.3389/fmicb.2017.02492)
Supplement: Table S3 — Thermal programs for PCR and qRT-PCR in this study. [file Table3.DOC]

**Table S3** Thermal programs for PCR and qRT-PCR in this study.

| Purpose | Thermal program in PCR |
| --- | --- |
| Cloning of *bcsmr1* | Initial denaturation: 94°C for 5 min; 34 cycles (94°C for 30 s, 57°C for 30s, 68°C for 1 min), final extension: 68°C for 10 min. |
| To determine expression of *bcpks12*, *bcpks13*, *bcygh1*, *bcbrn1*, *bcbrn2*, *bcscd1*, *bcsmr1* and *bcactA* by qRT-PCR | 95°C for 3 min (1 cycle), 95°C for 15 s, 59°C for 30s (39 cycles). Melt curve program: 95°C for 1 min (1 cycle), 65°C for 1 min (1 cycle), and a single temperature rise of 0.5°C held for 5s from 65°C to 95°C. |
| To confirm insertion of *bcsmr1*-BS and *bcsmr1*-OS in the plasmids pET28a-Bcsmr1-OS and pET28a-Bcsmr1-BS, respectively | Initial denaturation: 94°C for 2 min; 32 cycles: 94°C for 15 s, 68°C for 3 min, 68°C for 5 min and finally 16°C for 2 min. |
| Cloning of *bcsmr1* | Initial denaturation: 94°C for 5 min; 32 cycles: 94°C for 30 s, 58°C for 30s, 68°C for 3 min; final extension: 68°C for 10 min. |
| Cloning of the DNA sequence for the GAL4 activation domain | Initial denaturation: 94°C for 5 min; 32 cycles: 94°C for 30 s, 58°C for 30s, 68°C for 3 min; final extension: 68°C for 10 min. |
