# Supplementary material for: A Single-Nucleotide Deletion in the Transcription Factor Gene bcsmr1 Causes Sclerotial-Melanogenesis Deficiency in Botrytis cinerea
Source: Front Microbiol. 2017 Dec 12;8:2492. doi: 10.3389/fmicb.2017.02492 (PMC5733056; doi:10.3389/fmicb.2017.02492)
Supplement: Table S4 — The GenBank accession numbers for the melanin biosynthesis-related genes and their promoter regions in different isolates of B. cinerea. [file Table4.DOCX]

**Table S4**. The GenBank accession numbers for the melanin biosynthesis-related genes and their promoter regions in different isolates of *B. cinerea*.

| Isolate | GenBank Accession number | | | | | |
| --- | --- | --- | --- | --- | --- | --- |
|  | *bcpks12* | *bcpks13* | *bcbrn1* | *bcbrn2* | *bcscd1* | *bcsmr1* |
| OS isolates | | | | | | |
| XN-1 | KT266233 | KT266250 | KT266227 | KT266245 | KT266239 | KU743104 |
| S417 | KT266234 | KT726840 | KT726845 | KT726849 | KT266240 | KU743105 |
| T417 | KT266235 | KT726841 | KT726846 | KT726850 | KT266241 | KU743106 |
| BS isolates | | | | | | |
| WXt02-2 | KT266236 | KT726842 | KT726847 | KT726851 | KT266244 | KU743107 |
| HS016 | KT266237 | KT726843 | KT266228 | KT726852 | KT266242 | KU743108 |
| XN087 | KT266238 | KT726844 | KT726848 | KT726853 | KT266243 | KU743109 |
| B05.10^a^ | B0510_6611 | B0510_7826 | BC1G_04230.1 | B0510_7821 | B0510_7820 | Bcin02g08760 |

^a^The gene sequences for isolate B05.10 were downloaded from the public database in [Wageningen University](http://www.wageningenur.nl/) (<http://fungi.ensembl.org/Botrytis_cinerea>), whereas the gene sequences for other isolates were deposited in NCBI (<http://www.ncbi.nlm.nih.gov>).

**Table S5**. The GenBank accession numbers for the melanin biosynthesis-related genes and their promoter regions in different isolates of *B. cinerea*.

| Isolate | GenBank Accession number | | | | | |
| --- | --- | --- | --- | --- | --- | --- |
|  | *Bcpks12* | *Bcpks13* | *Bcbrn1* | *Bcbrn2* | *Bcscd1* | *Bcsmr1* |
| OS isolates | | | | | | |
| XN-1 | KT266233 | KT266250 | KT266227 | KT266245 | KT266239 | KU743104 |
| S417 | KT266234 | KT726840 | KT726845 | KT726849 | KT266240 | KU743105 |
| T417 | KT266235 | KT726841 | KT726846 | KT726850 | KT266241 | KU743106 |
| BS isolates | | | | | | |
| WXt02-2 | KT266236 | KT726842 | KT726847 | KT726851 | KT266244 | KU743107 |
| HS016 | KT266237 | KT726843 | KT266228 | KT726852 | KT266242 | KU743108 |
| XN087 | KT266238 | KT726844 | KT726848 | KT726853 | KT266243 | KU743109 |
| B05.10^a^ | B0510_6611 | B0510_7826 | BC1G_04230.1 | B0510_7821 | B0510_7820 | Bcin02g08760 |

^a^The gene sequences for isolate B05.10 were downloaded from the public database in [Wageningen University](http://www.wageningenur.nl/) (<http://fungi.ensembl.org/Botrytis_cinerea>), whereas the gene sequences for other isolates were deposited in NCBI (<http://www.ncbi.nlm.nih.gov>).
