# Supplementary material for: A Single-Nucleotide Deletion in the Transcription Factor Gene bcsmr1 Causes Sclerotial-Melanogenesis Deficiency in Botrytis cinerea
Source: Front Microbiol. 2017 Dec 12;8:2492. doi: 10.3389/fmicb.2017.02492 (PMC5733056; doi:10.3389/fmicb.2017.02492)
Supplement: Table S5 — Identity of bcsmr1 (open reading frame) and the polypeptide BcSMR1 encoded by bcsmr1 in black-colored sclerotial (or BS) or orange-colored sclerotial (or OS) isolates of Botrytis cinerea. [file Table5.DOC]

**Table S5** Identity of *bcsmr1* (open reading frame) and the polypeptide BcSMR1 encoded by *bcsmr1* in black-colored sclerotial (or BS) or orange-colored sclerotial (or OS) isolates of *Botrytis cinerea*.

| **Isolate** | **XN-1 (OS)** | **S417**  **(OS)** | **T417**  **(OS)** | **WXt02-2 (BS)** | **HS016**  **(BS)** | **XN087**  **(BS)** | **B05.10**  **(BS)** | **S59**  **(BS)** |
| --- | --- | --- | --- | --- | --- | --- | --- | --- |
| **XN-1 (OS)** |  | 100.0 | 100.0 | 99.1 | 99.6 | 98.9 | 99.4 | 99.96 |
| **S417 (OS)** | 100.0 |  | 100.0 | 99.1 | 99.6 | 98.9 | 99.4 | 99.96 |
| **T417 (OS)** | 100.0 | 100.0 |  | 99.1 | 99.6 | 98.9 | 99.4 | 99.96 |
| **WXt02-2 (BS)** | 99.8 | 99.8 | 99.8 |  | 99.6 | 99.9 | 99.5 | 99.82 |
| **HS016 (BS)** | 99.9 | 99.9 | 99.9 | 99.8 |  | 99.5 | 99.9 | 99.89 |
| **XN087 (BS)** | 99.8 | 99.8 | 99.8 | 99.9 | 99.8 |  | 99.4 | 99.79 |
| **B05.10 (BS)** | 99.8 | 99.8 | 99.8 | 99.8 | 99.9 | 99.7 |  | 99.86 |
| **S59 (BS)** | 99.78 | 99.78 | 99.78 | 99.47 | 99.89 | 99.36 | 99.79 |  |

(1) Length of the DNA sequences of the *bcsmr1* ORF: 3095 bp long in the OS isolates, whereas 3096 bp long in the BS isolates. Length of the amino acid sequences of BcSMR1: 465 aa long encoded by *bcsmr1* in the OS isolates, whereas 935 aa long encoded by *bcsmr1* in the BS isolates

(2) The percentages in the lower left corner (cells with white-colored background) and in the upper right corner (cells with gray-colored background) were calculated based on the nucleotides and the amino acids, respectively.

(3) The isolates XN-1, S417, T417, WXt02-2, HS016 and XN087 were serially assigned with GenBank accession numbers (*bcsmr1*) KU743104, KU743105, KU743106, KU743107, KU743108, KU743109. The GenBank accession number (*bcsmr1*) for isolate S59 is KX098785. The accession number for *bcsmr1* in isolate B05.10 is Bcin02g08760 ( http://fungi.ensembl.org/Botrytis_cinerea).
